# A Data-Driven Analysis of the Perceptual and Neural Responses to Natural Objects Reveals Organizing Principles of Human Visual Cognition

**DOI:** 10.1523/JNEUROSCI.1318-24.2024

**Published:** 2024-11-18

**Authors:** David M. Watson, Timothy J. Andrews

**Affiliations:** Department of Psychology and York Neuroimaging Centre, University of York, York YO10 5DD, United Kingdom

**Keywords:** data driven, fMRI, object perception, neural topography, visual cortex, visual perception

## Abstract

A key challenge in understanding the functional organization of the visual cortex stems from the fact that only a small proportion of the objects experienced during natural viewing can be presented in a typical experiment. This constraint often leads to experimental designs that compare responses to objects from experimenter-defined stimulus conditions, potentially limiting the interpretation of the data. To overcome this issue, we used images from the THINGS initiative, which provides a systematic sampling of natural objects. A data-driven analysis was then applied to reveal the functional organization of the visual brain, incorporating both perceptual and neural responses to these objects. Perceptual properties of the objects were taken from an analysis of similarity judgments, and neural properties were taken from whole-brain fMRI responses to the same objects. Partial least squares regression (PLSR) was then used to predict neural responses across the brain from the perceptual properties while simultaneously applying dimensionality reduction. The PLSR model accurately predicted neural responses across the visual cortex using only a small number of components. These components revealed smooth, graded neural topographies, which were similar in both hemispheres and captured a variety of object properties including animacy, real-world size, and object category. However, they did not accord in any simple way with previous theoretical perspectives on object perception. Instead, our findings suggest that the visual cortex encodes information in a statistically efficient manner, reflecting natural variability among objects.

## Significance Statement

The ability to recognize objects is fundamental to how we interact with our environment, yet the organizing principles underlying neural representations of visual objects remain contentious. In this study, we sought to address this question by analyzing perceptual and neural responses to a large, unbiased sample of objects. Using a data-driven approach, we leveraged perceptual properties of objects to predict neural responses using a small number of components. This model predicted neural responses with a high degree of accuracy across the visual cortex. The components did not directly align with previous explanations of object perception. Instead, our findings suggest the organization of the visual brain is based on the statistical properties of objects in the natural world.

## Introduction

The ability to perceive and recognize objects in our environment is crucial for guiding behavior (Peelen and Downing, 2017; Bracci and Op de Beeck, 2023). Visual areas involved in object perception are organized along a ventral processing pathway extending from low-level regions in the occipital lobe to high-level regions in the inferior temporal lobe (Milner and Goodale, 1995; Grill-Spector and Weiner, 2014). In low-level regions, neural representations of objects are tightly linked to simple image features (Hubel and Wiesel, 1968; Wandell et al., 2007). However, in higher-level stages, neural representations are linked to more complex object properties (Grill-Spector and Weiner, 2014; Bracci and Op de Beeck, 2023). Nevertheless, the organizing principles underlying the complexity in high-level visual processing remain a topic of ongoing debate.

High-level visual cortex has traditionally been characterized by category-selective regions that show preferences for specific visual object categories, including places (Epstein and Kanwisher, 1998), faces (Kanwisher et al., 1997), bodies (Downing et al., 2001), visual word forms (Cohen et al., 2000), inanimate objects (Malach et al., 1995), and food (Khosla et al., 2022). However, it has also been proposed that more basic organizing principles may underlie these neural responses (Grill-Spector and Weiner, 2014). Evidence supporting this hypothesis includes findings that neural responses can be explained by properties such as animacy (Chao et al., 1999; Kriegeskorte et al., 2008b; Konkle and Caramazza, 2013), real-world size (Konkle and Oliva, 2012), and low- and mid-level visual properties of objects (Levy et al., 2001; Andrews et al., 2015; Long et al., 2018). While these principles are not mutually exclusive, the integration of different properties within the visual brain remains unclear. One challenge in addressing this issue is that only a small proportion of the objects we normally experience in natural viewing can be shown during a typical experiment, leading to an uneven sampling of objects. This may introduce biases and constrain our understanding of the underlying functional organization.

Data-driven methods provide an alternative approach to exploring the functional organization of the brain (Cunningham and Yu, 2014; Watson et al., 2017; Brunton and Beyeler, 2019; Coggan et al., 2019). For example, Hebart et al. (2020) applied a data-driven approach to elucidate dimensions underpinning the perception of objects from the THINGS database, which provides a large and unbiased sampling of natural objects (Hebart et al., 2019). They identified 66 stimulus dimensions that explained perceptual representations of the objects. Recently, the same group used a linear encoding model to predict neural responses from these 66 stimulus dimensions, demonstrating sparse and distributed representations of the dimensions across the visual cortex (Contier et al., 2024). Although this indicates these stimulus dimensions can explain variance in the neural responses, it remains unclear whether the visual brain can be explained by a smaller number of organizing principles.

In this study, we employed partial least squares regression (PLSR) to investigate whether a low-dimensional representation of natural objects can be derived by integrating both perceptual and neural properties. PLSR assumes the population-level neural responses can be explained by a reduced set of hidden or latent variables (Krishnan et al., 2011; Cunningham and Yu, 2014). Our aim was to determine whether the representation of objects can be captured by a simplified model, based on a small number of latent components revealing key dimensions of variation among natural stimuli. We leveraged publicly available data from the THINGS initiative (Hebart et al., 2019, 2023), comprising behavioral and neural responses to images of objects in natural contexts. We found the latent components predicted neural responses throughout low- and high-level visual cortices with a high degree of accuracy. There was also a smooth and graded change in selectivity to the components, suggesting continuous map-like representations of information, similar to those which characterize early sensory areas. Moreover, the representations were similar in both hemispheres. Together, these results indicate the components identified by this data-driven approach reflect fundamental organizing principles of the visual brain.

## Material and Methods

### Dataset

All data were obtained from the publicly available THINGS initiative (https://things-initiative.org/; Hebart et al., 2019). This database includes images and associated datasets for 1,854 object concepts. We first obtained a data-driven stimulus model derived from behavioral similarity judgments of these object concepts (Hebart et al., 2020). In brief, crowd-sourcing was used to obtain millions of odd-one-out judgments on triplets of object images. Computational modeling was used to derive a low-dimensional feature space, such that the similarity between representations of object concepts within this space was predictive of the corresponding human similarity judgments. Dimensions of this space correspond to meaningful properties of the object concepts and span low-, mid-, and high-level features. We used an updated version of this model comprising 66 stimulus dimensions derived from ∼4.7 million trials (Hebart et al., 2023). Each of the 1,854 object concepts is thus represented as a sample within this 66-dimensional space. We then selected the 720 samples within this space corresponding to the object concepts included in the MRI dataset.

We obtained fMRI data comprising neural responses to 12 images from each of 720 object concepts (8,640 unique images) in three subjects (Hebart et al., 2023). We obtained single-trial parameter estimates calculated by the THINGS initiative. In brief, fMRI data were preprocessed using *fMRIprep* (Esteban et al., 2019), including slice-timing correction, head motion correction, susceptibility distortion correction using field maps, ICA denoising, and alignment to each subject's T1-weighted anatomical scan. Single-trial parameter estimates were then derived using a procedure modified from the *GLMsingle* approach (Prince et al., 2022). We additionally reconstructed each subject's cortical surfaces from their T1- and T2-weighted anatomical scans using FreeSurfer 6.0 (Dale et al., 1999). The single-trial parameter estimates were transformed to each subject's cortical surface and then further to the *fsaverage5* surface (comprising 10,242 vertices per hemisphere) via a surface-based registration (Fischl et al., 1999). The lower resolution *fsaverage5* surface was chosen to ensure computational tractability for the neural encoding analyses (described below). Surface-based spatial smoothing was then applied at FWHM = 4 mm (twice the voxel resolution). The parameter estimates were then averaged over the 12 images within each of the 720 object concepts and then further averaged over the three subjects. These averaged parameter estimates were then restricted to the cortical vertices (excluding the medial wall) and concatenated over hemispheres.

The 720 objects concepts are therefore represented as samples within each of two feature spaces: (1) the stimulus model comprising 66 dimensions and (2) the neural space comprising the group average responses measured over the ∼20,000 cortical vertices in both hemispheres.

### Neural encoding model

**Figure 1.**
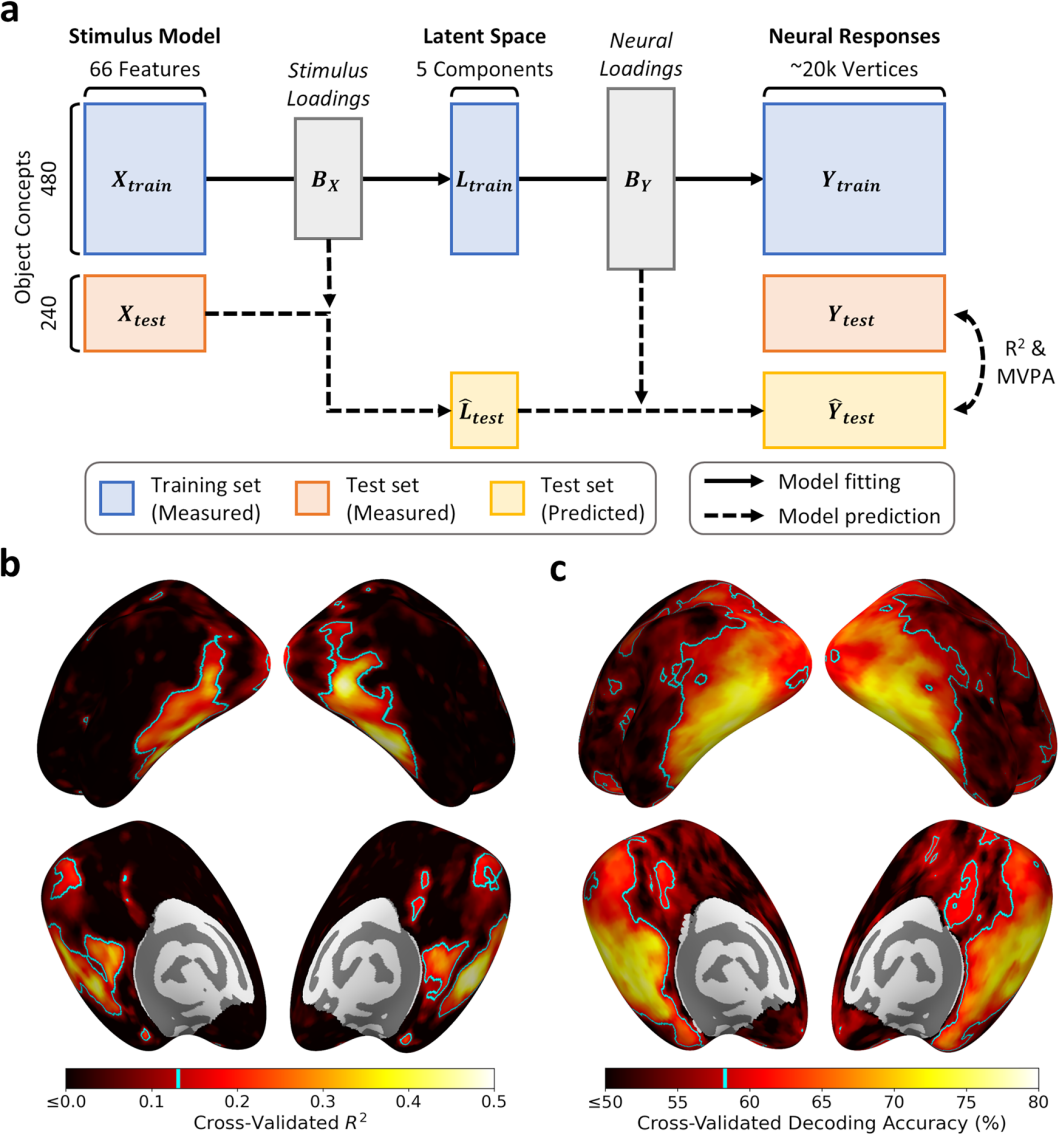
Neural encoding model and prediction accuracies. ***a***, Schematic of neural encoding model. The 480 object concepts in the training set are measured by the stimulus model $\left(X_{\mathrm{train}}\right)$ and whole-brain neural responses $\left(Y_{\mathrm{train}}\right)$. Partial least squares regression (PLSR) derives a mapping from the stimulus model to the neural responses via a low-dimensional (*k *= 5) latent space $\left(L_{\mathrm{train}}\right)$. Components of the latent space are linear combinations of both the stimulus model and neural features and are defined to maximize prediction of the neural responses. The 240 object concepts in the test set are projected from the stimulus model to the neural space using the PLSR coefficients. The predicted and measured neural responses within the test set are compared to assess prediction accuracy. ***b***, Cross-validated *R*^2^ indicates the variance explained in univariate responses across object concepts. ***c***, Cross-validated decoding accuracy for pairwise classifications between object concepts based on multivariate responses within surface searchlights. Cyan highlights indicate the significance threshold (*p *< 0.05, FWER corrected) determined by permutation testing. See Extended Data Figure 1-1 for a grid search illustrating performance over different numbers of components and Extended Data Figure 1-2 for locations of scene- and face-selective regions.

We next adopted a neural encoding approach to predict the neural responses from the stimulus model representations (Fig. 1*a*). We used PLSR to incorporate dimensionality reduction into the encoding model. This inserts an additional lower-dimensional latent space between the stimulus model and neural spaces. The PLSR then derives two sets of loadings—first mapping between the latent space and stimulus model and second mapping between the latent space and neural responses. Each component in the latent space represents a linear combination of the features in both the stimulus model and neural spaces. The components are ordered by their predictive power of the neural responses, so that the first component is maximally predictive of the neural responses, the second component is second most predictive, and so forth. We adopted a cross-validated approach, so that the 720 object concepts were split into a training set comprising the first 480 concepts and a test set comprising the final 240 concepts. Samples in both the stimulus model and neural spaces were *z*-scored along each feature for the training and test sets independently, such that the PLSR loadings are derived in standardized units. The PLSR was fit to the 480 concepts in the training set.

10.1523/JNEUROSCI.1318-24.2024.f1-1 Figure 1-1 Grid search of prediction accuracy over varying numbers of PLSR components to retain. A 5-fold cross-validation was nested within the 480 object concepts of the main training set. For each fold of the cross-validation, a PLSR model was fit to the inner training set, retaining between 1 and 10 components, then whole-brain R^2^ maps were calculated for the inner test set. A summary statistic was calculated by taking the maximum R^2^ value over all vertices, indicating the peak prediction accuracy over the whole brain. The graph illustrates the mean and standard deviation (over the 5 cross-validation folds) of these maximal R^2^ values for each number of components. Prediction accuracy plateaus between 4 and 6 components. Download Figure 1-1, TIF file.

**Figure 2.**
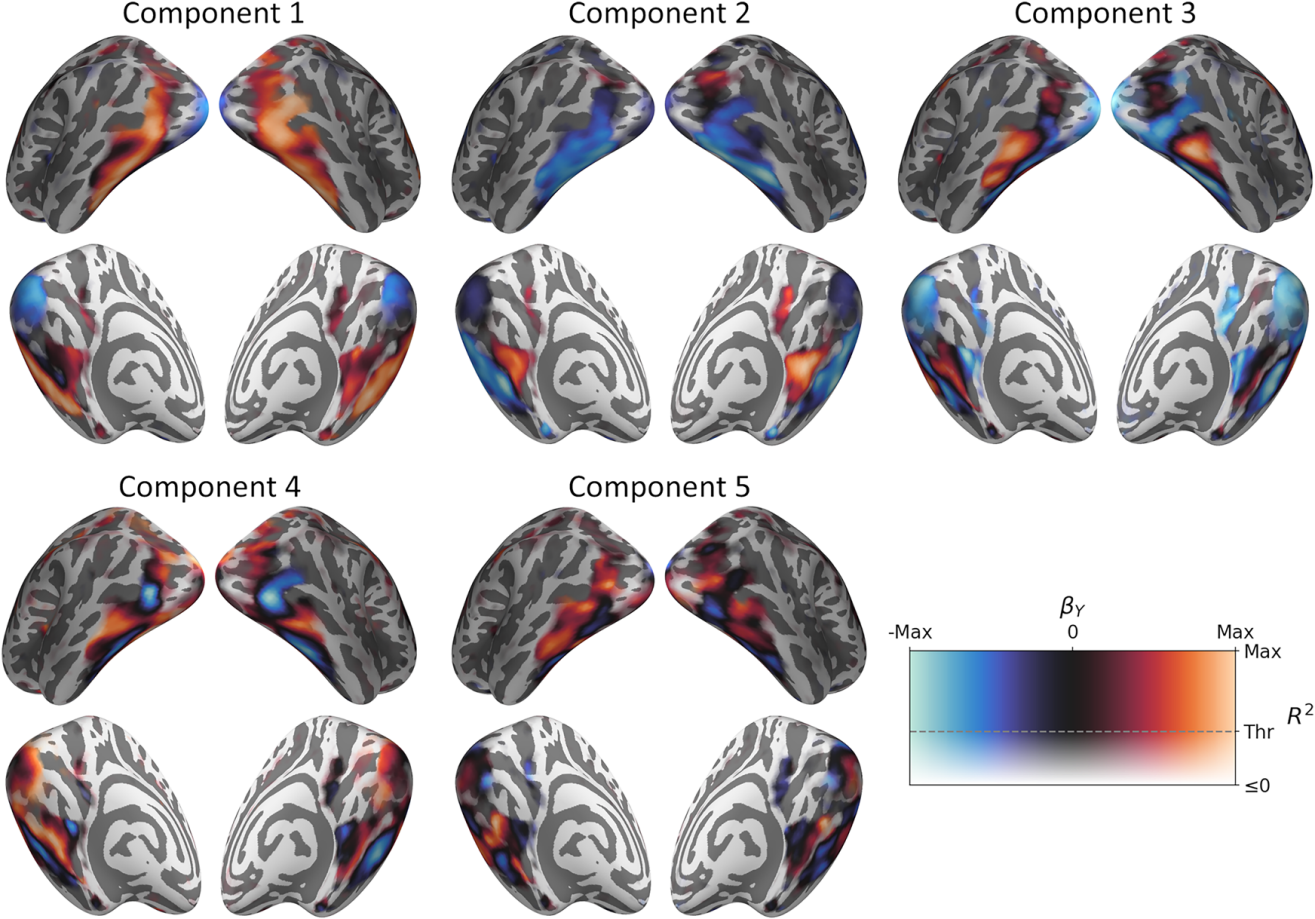
Neural loadings of each PLSR component. Object concepts that score more positively or negatively along each component elicit stronger responses in regions highlighted in red and blue, respectively. The transparency of the color map is modulated by the cross-validated *R*^2^ map (compare Fig. 1*b*) so that vertices below threshold appear with increasing transparency.

10.1523/JNEUROSCI.1318-24.2024.f1-2 Figure 1-2 Locations of core scene- and face-selective regions of interest. Regions are defined from the group average contrast of “scenes > faces” using the category localiser task data. Scene regions: Parahippocampal Place Area (PPA), Retrosplenial Complex (RSC), Occipital Place Area (OPA). Face regions: Fusiform Face Area (FFA), Occipital Face Area (OFA). Download Figure 1-2, TIF file.

The number of components to retain was determined by a cross-validated grid search nested within the main training set (Extended Data Fig. 1-1). Prediction accuracy plateaued between four and six components; we therefore chose to retain five components. Importantly, the components are ordered, so while retaining more components may alter prediction accuracy at the cost of increasing model complexity, it would not affect the earlier components already obtained. The stimulus model is thus represented by a 480-by-66 matrix $\left(X_{\mathrm{train}}\right)$, while the neural space is represented by an approximately 480-by-20,000 matrix $\left(Y_{\mathrm{train}}\right)$. Similarly, the latent space is represented by a 480-by-5 matrix $\left(L_{\mathrm{train}}\right)$. The PLSR derives: (1) a 66-by-5 matrix of loadings $\left(B_{X}\right)$ mapping between the latent space and stimulus model and (2) an approximately 20,000-by-5 matrix of loadings $\left(B_{Y}\right)$ mapping between the latent space and neural responses. The neural encoding analysis was implemented using *scikit-learn* (Pedregosa et al., 2011).

To provide additional descriptives of the components, we obtained ratings for 12 object properties provided with the THINGSplus metadata (Stoinski et al., 2023). These include ratings for the object size and how natural, living, moving, manmade, precious, heavy, graspable, holdable, movable, pleasant, and arousing the objects are. Object size was rated on a 520-point continuous scale, with higher ratings indicating larger objects. The remaining properties were rated on 7-point Likert scales. We used an updated version of the arousal rating task which emphasized that the construct should represent a scale between “arousing” versus “calming.” Full details are provided in Stoinski et al. (2023). We obtained mean ratings at the concept-level for each of the 480 object concepts in the training set. We then computed Spearman correlations between ratings on each of the 12 object properties and latent scores along each of the five components (matrix $L_{\mathrm{train}}$), applying a Holm–Bonferroni correction for multiple comparisons (Holm, 1979).

### Cross-validated prediction accuracies

We assessed the prediction accuracy of the model using the 240 object concepts within the test set. The PLSR coefficients derived from the training set were applied to the stimulus model $\left(X_{\mathrm{test}}\right)$ to predict the corresponding neural responses $\left({\hat{Y}}_{\mathrm{test}}\right)$. These predicted responses were then compared with the measured neural responses $\left(Y_{\mathrm{test}}\right)$ to assess the cross-validated predication accuracy. We adopted two approaches for this comparison.

First, we measured the coefficient of determination (*R*^2^), representing the proportion of variance explained in the univariate responses over the 240 object concepts at each vertex (Fig. 1*b*). We determined statistical significance using a maximum statistic permutation test. On each permutation, we randomized the order of the training samples in the stimulus model space and refit the PLSR model. This was then used to predict neural responses from the stimulus model representation of the unpermuted test set, such that the model was fit to permuted data but tested against unpermuted data. The whole-brain *R*^2^ map was calculated and the largest *R*^2^ value over all vertices was retained. This process was repeated for 1,000 permutations to produce an empirical null distribution that can be applied to the whole brain while controlling the familywise error rate. The 95th percentile of this distribution provides the threshold for statistical significance (*p *< 0.05, FWER corrected).

Second, we employed a multivariate pattern analysis (MVPA) to test pairwise decoding between predicted and measured patterns of neural response across the 240 object concepts (Fig. 1*c*). We used a surface-based searchlight (Kriegeskorte et al., 2006) with 8 mm radius discs (yielding a median of 28 vertices per searchlight) applied along the mid-thickness cortical surface. This analysis was implemented using the *CoSMoMVPA* (https://www.cosmomvpa.org/; Oosterhof et al., 2016) and *surfing* (https://github.com/nno/surfing; Oosterhof et al., 2011) MATLAB toolboxes. In each searchlight, we computed pairwise correlations between the predicted and measured response patterns to the 240 object concepts. This yielded a 240-by-240 asymmetric matrix of correlations (R), with predicted and measured responses represented across rows and columns, respectively. We next applied *k*-nearest neighbor (*k *= 1) classification to decode between pairwise combinations of object concepts. For a given target concept (i) and reference concept (j), we tested if the predicted responses to the target concept were more strongly correlated with the measured responses to the target concept than to the measured responses to the reference concept. This corresponds to stepping through the rows of matrix R and testing if the on-diagonal element is greater than each of the other elements in that row. This derives a 240-by-240 matrix of binary values (A), such that $A_{i,j}=R_{i,i}>R_{i,j}=r({\hat{y}}_{i},y_{i})>r({\hat{y}}_{i},y_{j})$, where ${\hat{y}}_{n}$ and $y_{n}$ represent the predicted and measured response patterns to a given object concept (n), respectively. Note that the target and reference concepts will be reversed in symmetrically opposite elements of this matrix. The proportion decoding accuracy is given by the mean of the off-diagonal values in this matrix. This value was assigned into the central vertex of the searchlight, and the process then repeated for all searchlights to obtain a whole-brain map of decoding accuracies. To determine statistical significance, we again adopted a maximum statistic permutation test. On each permutation, we shuffled the order of the object concepts for the measured responses (columns of the correlations matrix). We recomputed the whole-brain searchlight analysis and recorded the highest decoding accuracy over all searchlights. We repeated this for 1,000 permutations to obtain an empirical null distribution. The 95th percentile of this distribution provides the threshold for statistical significance (*p *< 0.05, FWER corrected).

### GLM analysis

To test the reproducibility of the PLSR neural component loadings $\left(B_{Y}\right)$, we conducted an additional univariate general linear model (GLM) analysis using the 240 object concepts in the test set. First, the PLSR model fit to the training set was used to project the test concepts from the stimulus model space into the latent space. This produces a 240-by-5 matrix of predicted latent scores $\left({\hat{L}}_{\mathrm{test}}\right)$ for the test set. These latent scores were then demeaned along each component and used as a design matrix within a GLM analysis to predict the measured neural responses in the test set $\left(Y_{\mathrm{test}}\right)$. The GLM analysis was implemented using the *mri_glmfit* command in FreeSurfer (Dale et al., 1999). This produces a new matrix of GLM regression coefficients the same shape as the PLSR neural component loadings matrix $\left(B_{Y}\right)$. This allows testing the relationship between the predicted latent scores and measured neural responses for the held-out object concepts in the test set.

### Comparing neural loadings

We compared the symmetry of the PLSR neural component loadings (derived from the training set) between hemispheres. The component loadings and cross-validated *R*^2^ maps were resampled to the *fsaverage_sym* surface, which provides a symmetrical representation of the *fsaverage* brain. The right hemisphere maps were then mirrored onto the left hemisphere. A region of interest was generated from the intersection of the thresholded *R*^2^ maps on the left and mirror-right hemispheres. The component loadings were then restricted to this region and correlated within and between the left and mirror-right hemispheres.

We also compared the PLSR neural component loadings (derived from the training set) and the regression loadings from the GLM analysis of the test set. Both sets of loadings were restricted to the thresholded cross-validated *R*^2^ maps and concatenated over hemispheres. The loadings were then correlated between the PLSR and GLM analyses.

### DCNN modeling

We conducted a series of representational similarity analyses (RSAs; Kriegeskorte et al., 2008a) comparing representations of object concepts within the PLSR latent space to representations within layer activations of two deep convolutional neural networks (DCNNs). We obtained versions of AlexNet (Krizhevsky et al., 2017) and VGG16 (Simonyan and Zisserman, 2014), implemented in MATLAB, pretrained for object classification on the ImageNet dataset. Images from the THINGS database for each of the 480 object concepts in the training set were passed through these DCNNs, and the activations were extracted for all convolutional and fully connected layers. The activations were then averaged over images within each object concept. For each layer, the averaged activation vectors were used to compute representational dissimilarity matrices (RDMs) measuring the pairwise correlation distances between the 480 object concepts.

We next obtained corresponding RDMs for the PLSR model by measuring the pairwise correlation distances between object concepts using their latent scores over the five components (Fig. 1*a*, matrix $L_{\mathrm{train}}$). We also computed PLSR RDMs for each component individually by measuring the pairwise absolute differences in latent scores between object concepts along a given component. The PLSR RDMs (for the full space and for each component) were then correlated with the RDMs for each DCNN layer. Statistical significance of the RSA correlations was determined using a maximum statistic permutation test. On each permutation, the order of the object concepts was randomly permuted (corresponding to shuffling the rows and columns of the RDMs). The RSA correlations were then recomputed for all DCNN layers (and PLSR components, if applicable), and the maximum correlation over all comparisons was recorded. This process was repeated for 10,000 permutations to obtain an empirical null distribution. The 95th percentile of this distribution provides the threshold for statistical significance (*p *< 0.05) controlling the familywise error rate over DCNN layers (and PLSR components, if applicable).

## Results

### Reliability of the PLSR components

We analyzed behavioral and neural responses to 720 unique object concepts from the THINGS initiative (Hebart et al., 2019). The behavioral responses were described by a stimulus model derived from a data-driven analysis, in which each object concept is represented by 66 stimulus dimensions (Hebart et al., 2020). The neural space is based on the group average responses to each object concept measured over the ∼20,000 cortical vertices in both hemispheres (Hebart et al., 2023). We employed a neural encoding model, utilizing PLSR, to predict the neural responses from the stimulus model, retaining the first five components of the latent space (Fig. 1*a*).

To test the reliability of the PLSR model, we fit the model to 480 of the 720 object concepts and then tested its performance at predicting the neural responses to the remaining 240 concepts. Prediction accuracy was assessed by comparing the predicted and measured neural responses within the test set. Figure 1*b* shows whole-brain *R*^2^ maps, representing the proportion of variance explained in the univariate responses over the 240 object concepts at each vertex. This highlighted many regions of the visual brain. The highest prediction accuracies (up to ∼48% variance explained) were observed in the ventral visual stream, though significant accuracies also extended into early visual, lateral occipital, and retrosplenial regions. For comparison, the locations of scene- and face-selective regions are illustrated in Extended Data Figure 1-2.

As a further measure of model performance, we used a MVPA to classify object concepts from patterns of neural response over local patches of the cortical surface. A *k*-nearest neighbor classifier decoded between pairwise combinations of object concepts in the test set based on the correlation between measured and predicted response patterns. For each object concept, we performed a series of binary classifications testing whether the predicted response patterns to that concept were more similar to the measured response patterns to that same concept versus each of the other concepts in turn. Overall decoding accuracy was defined as the average performance over all pairwise binary classifications. A surface-based searchlight derived a whole-brain map of decoding accuracies (Fig. 1*c*). This highlighted a more expansive set of regions than the univariate analysis. The highest decoding accuracies were again observed in the ventral visual cortex (up to ∼75% accuracy), but significant decoding also extended into early visual, lateral occipital, retrosplenial, and dorsal parietal regions. Thus, both univariate and multivariate measures indicate the PLSR neural encoding model predicted neural responses to object concepts in the held-out test set with a high degree of accuracy in visual areas of the brain.

### Topographic neural representations of PLSR components

We next inspected the representation of each component in the brain. Figure 2 illustrates the neural component loadings (Fig. 1*a*, matrix $B_{Y}$)—object concepts that score more positively or negatively along a given component produce stronger responses in brain regions highlighted in red and blue, respectively. Each component revealed representations that showed a continuous graded change across the visual brain and a high degree of similarity across the hemispheres.

Component 1 showed an anterior-posterior gradient, which was positively associated with high-level visual regions in the ventral stream and negatively associated with early visual regions in the occipital lobe. Component 2 revealed a medial-lateral gradient, which was positively and negatively associated with medial and lateral aspects of the ventral visual cortex, respectively. Positive loadings aligned well with the core scene-selective regions (parahippocampal place area, retrosplenial complex, and occipital place area; compare Extended Data Fig. 1-2).

Component 3 indicated a repeating medial-to-lateral pattern across the ventral stream. Negative loadings were located along the medial aspect of the collateral sulcus, transitioning to positive loadings along the lateral aspect of the collateral sulcus and medial aspect of the fusiform gyrus. Loadings then transitioned back to negative along the lateral aspect of the fusiform gyrus and medial aspect of the lateral occipitotemporal sulcus, further extending posterior and superior into the inferior occipital gyrus and sulcus and middle occipital gyrus along the lateral occipital surface. Loadings transitioned back to positive along the lateral aspect of the lateral occipitotemporal sulcus and medial aspect of the inferior temporal gyrus. Positive loadings were also located along more superior aspects of the lateral occipital surface, extending through the middle occipital sulcus.

Component 4 also revealed a repeating medial-to-lateral pattern. This appeared similar to component 3 but included a greater representation of medial positive loadings extending into the fusiform gyrus and a greater representation of lateral negative loadings extending into the lateral occipitotemporal sulcus. Furthermore, component 3 was negatively associated with early visual regions, whereas component 4 exhibited positive loadings. Component 5 similarly indicated a repeating medial-lateral pattern, but also included posterior-anterior elements, and appeared less symmetric between the hemispheres than previous components. For instance, the left hemisphere included negative loadings in posterior and anterior aspects of the fusiform gyrus, with positive loadings in between.

### Similarity of neural representations

**Figure 3.**
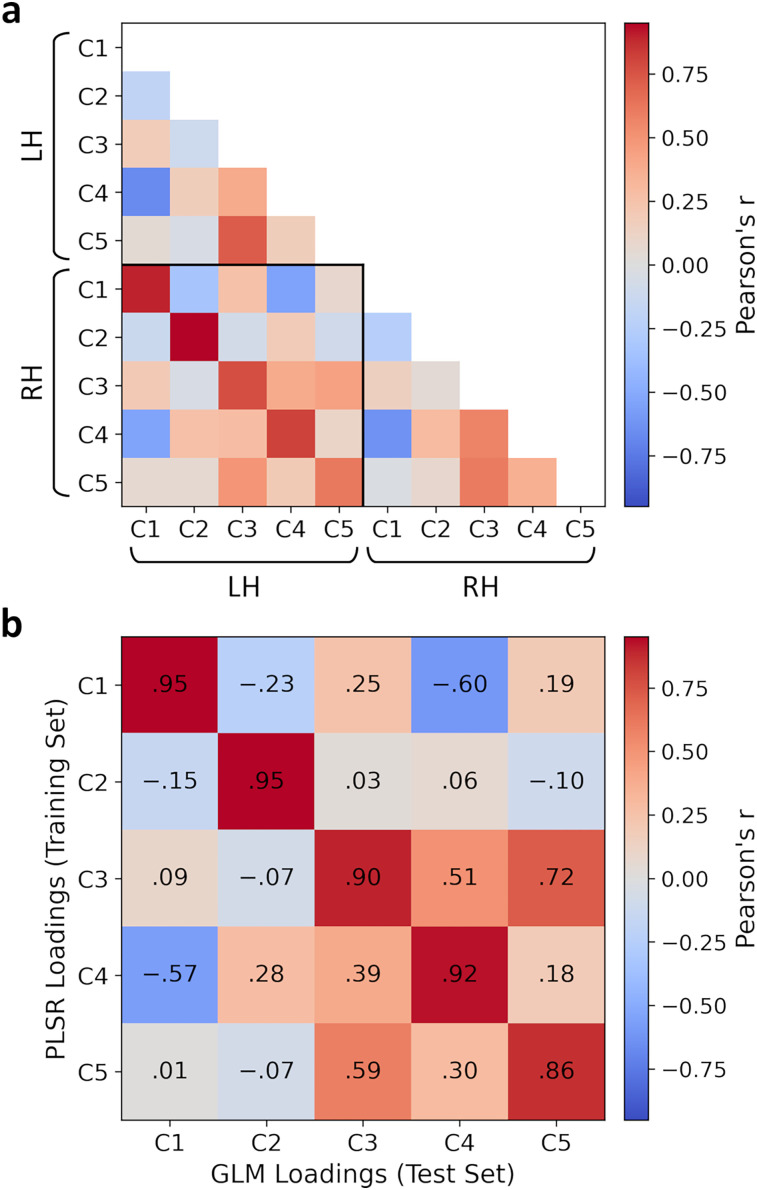
Similarity of components from the PLSR model. ***a***, Correlations of PLSR neural component loadings within and between hemispheres, restricted to the overlap of the thresholded cross-validated *R*^2^ maps in each hemisphere. ***b***, Correlations between PLSR and GLM neural component loadings, concatenated over hemispheres and restricted to thresholded cross-validated *R*^2^ maps.

We next compared the spatial similarity of the PLSR neural component loadings within and between hemispheres. To compare between hemispheres, loadings for the right hemisphere were mirrored onto the left hemisphere. Figure 3*a* shows correlations of the loadings between components (within and between hemispheres) for above-threshold vertices. Correlations appeared high between corresponding components in each hemisphere (demonstrated by the strong diagonal within the between-hemisphere square in the correlation matrix), indicating a high degree of symmetry between hemispheres. There was also a good degree of symmetry in the correspondence between components; for instance, components 1 and 2 appeared anticorrelated both within and between hemispheres.

**Figure 4.**
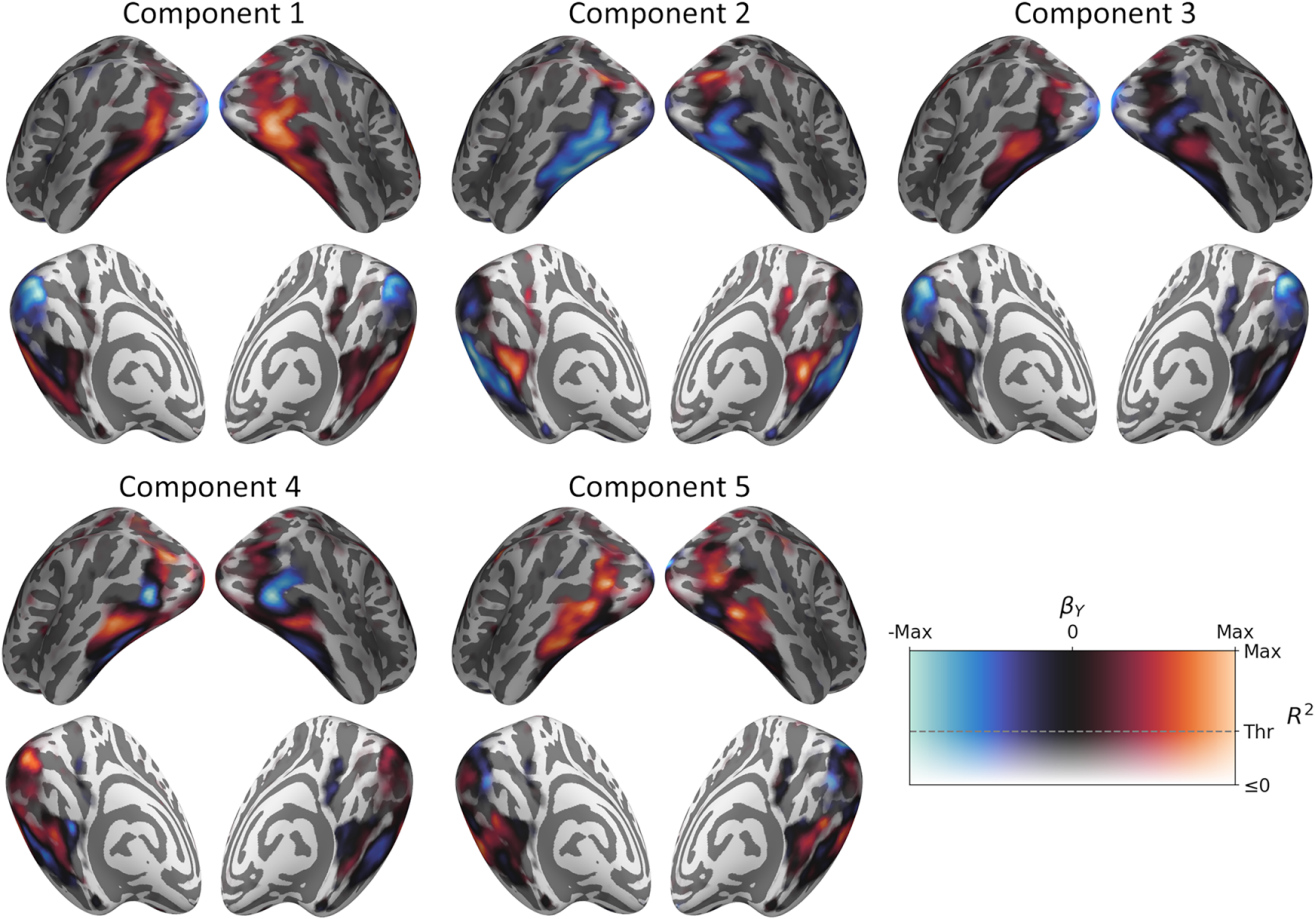
Neural loadings of each GLM component. The PLSR fit to the training set is used to project object concepts in the test set from the stimulus model to the latent space. A GLM then maps these predicted latent scores to the measured neural responses for the test set. Object concepts that score more positively or negatively along each component elicit stronger responses in regions highlighted in red and blue, respectively. The transparency of the color map is modulated by the cross-validated *R*^2^ map (compare Fig. 1*b*) so that vertices below threshold appear with increasing transparency.

To test the reproducibility of the neural patterns, we conducted a GLM analysis of the 240 object concepts in the test set. Object concepts were projected from the stimulus model to the latent space using the PLSR model fit to the training set. The GLM then mapped these predicted latent scores to the measured neural responses. The resulting GLM loadings are illustrated in Figure 4 and revealed a striking resemblance to the PLSR neural component loadings derived from the training set (compare Fig. 2). Figure 3*b* shows the spatial correlations between PLSR and GLM loadings for above-threshold vertices. Within-component correlations were very high, indicating a strong similarity between the PLSR loadings derived from the training set and the GLM loadings derived from the test set. There was also a high degree of symmetry over the diagonal of the matrix, further indicating a good degree of consistency in the relationships between components. Thus, the topographic representations associated with all five components were highly reproducible between independent splits of the data.

### Representation of object properties on PLSR components

**Figure 5.**
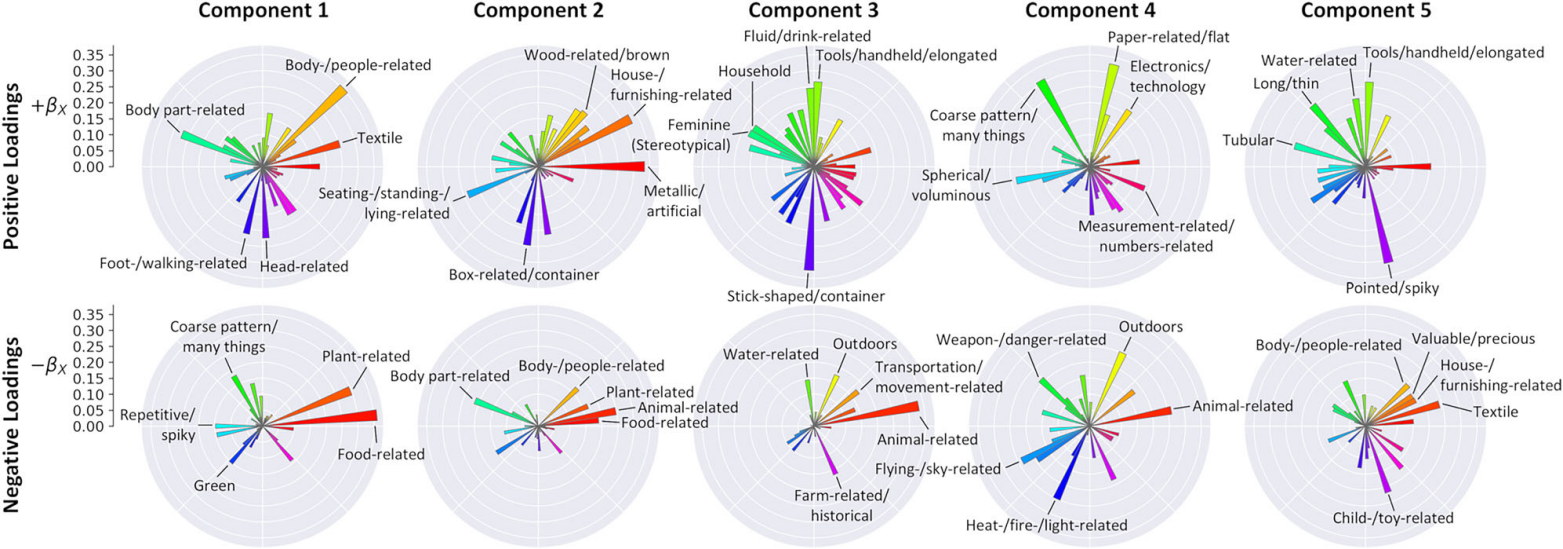
Polar plots displaying stimulus model loadings for each PLSR component. Positive loadings are shown in the top row, while negative loadings are shown in the bottom row. Stimulus model dimensions are mapped around the polar axis. The radial axis indicates the magnitude of the loadings. Annotations indicate top and bottom five stimulus dimensions loading on each component.

**Figure 6.**
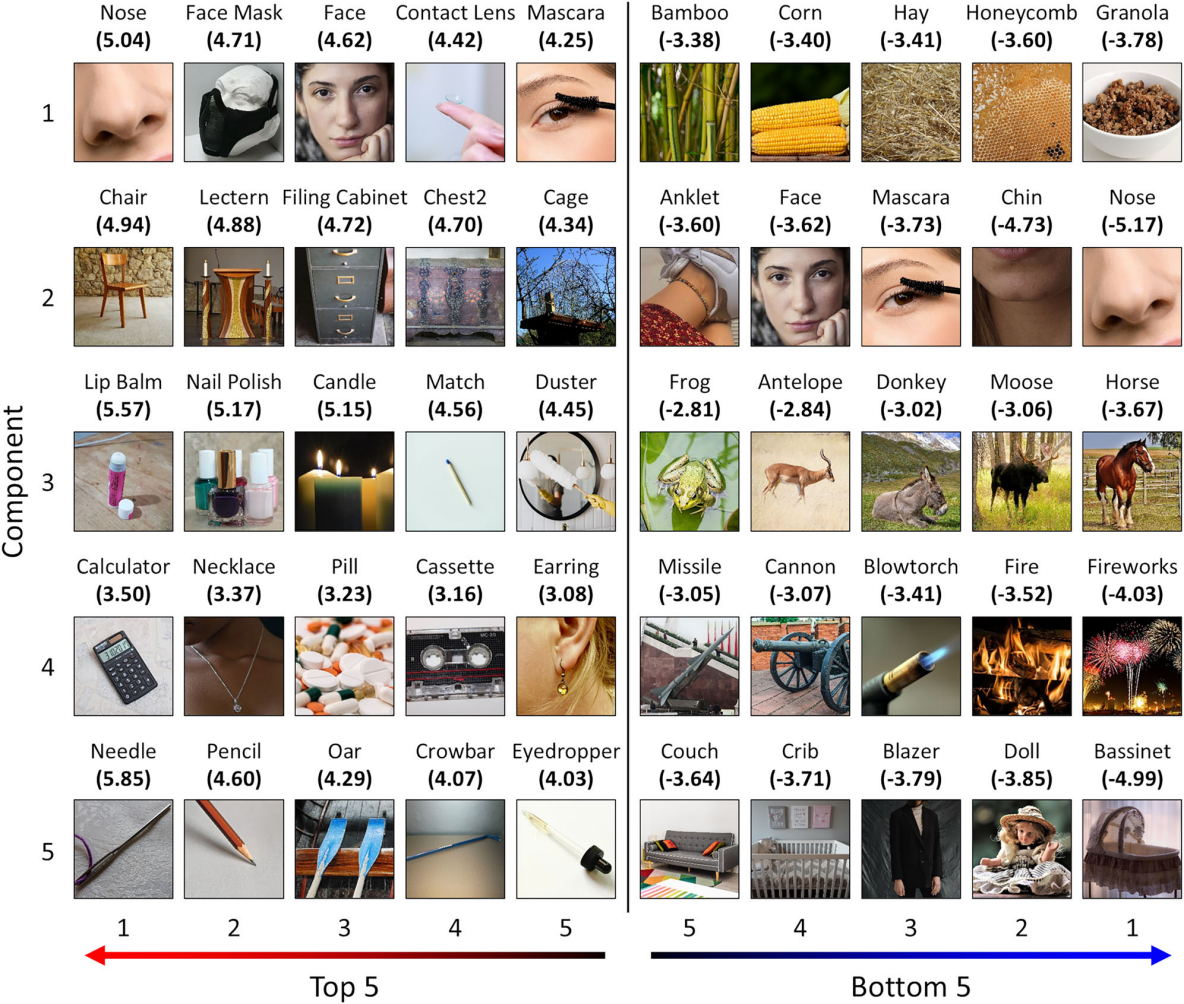
Top and bottom five object concepts (within the training set) scored along each PLSR component. Representative images are illustrated for each concept, and scores are indicated in parentheses. Images in this figure have been replaced with images from the THINGSplus dataset distributed under a CC0 license, which are representative of each object concept. See Extended Data Figures 6-1–6-5 for expanded lists of the top, middle, and bottom 10 object concepts scored along each component.

We next inspected how the stimulus model features and objects concepts from the behavioral analysis (Hebart et al., 2020) relate to each PLSR component. First, the stimulus loadings (Fig. 1*a*, matrix $B_{X}$) show how the stimulus features obtained from the behavioral analysis relate to the latent components. Table 1 shows the top and bottom five stimulus model dimensions loading on each component. This indicates the stimulus dimensions most strongly associated with the positive and negative ends of each component. Expanded lists of the top, middle, and bottom 10 stimulus model loadings on each component are provided in Extended Data Tables 1-1–1-5. The polar plots in Figure 5 provide an alternative visualization of the stimulus model loadings—these highlight the different distributions of loadings across each component. Second, the object concepts in the training set were projected from the stimulus model to the latent space (Fig. 1*a*, matrix $L_{\mathrm{train}}$). Figure 6 illustrates the top and bottom five object concepts scored on each component. Expanded lists of the top, middle, and bottom 10 object concepts scored on each component are illustrated in Extended Data Figures 6-1–6-5.

**Table 1.** Top and bottom five stimulus model dimensions loading on each PLSR component

	Component				
1	2	3	4	5
Top 5	1	Body-/people-related (0.35)	Metallic/artificial (0.33)	Stick-shaped/container (0.33)	Paper-related/flat (0.33)	Pointed/spiky (0.31)
	2	Body part-related (0.27)	House-/furnishing-related (0.33)	Tools/handheld/elongated (0.27)	Coarse pattern/many things (0.31)	Tools/handheld/elongated (0.26)
	3	Textile (0.25)	Box-related/container (0.25)	Fluid/drink-related (0.25)	Spherical/voluminous (0.23)	Long/thin (0.25)
	4	Head-related (0.22)	Seating-/standing-/lying-related (0.24)	Feminine (stereotypical) (0.23)	Electronics/technology (0.21)	Tubular (0.23)
	5	Foot-/walking-related (0.22)	Wood-related/brown (0.22)	Household (0.22)	Measurement-/numbers-related (0.18)	Water-related (0.21)
Bottom 5	5	Repetitive/spiky (−0.15)	Plant-related (−0.17)	Water-related (−0.15)	Weapon-/danger-related (−0.21)	Valuable/precious (−0.17)
	4	Green (−0.15)	Body-/people-related (−0.17)	Farm-related/historical (−0.16)	Flying-/sky-related (−0.24)	House-/furnishing-related (−0.18)
	3	Coarse pattern/many things (−0.18)	Food-related (−0.19)	Outdoors (−0.17)	Heat-/fire-/light-related (−0.25)	Body-/people-related (−0.18)
	2	Plant-related (−0.30)	Body part-related (−0.21)	Transportation-/movement-related (−0.17)	Outdoors (−0.25)	Child-/toy-related/cute (−0.22)
	1	Food-related (−0.36)	Animal-related (−0.25)	Animal-related (−0.33)	Animal-related (−0.26)	Textile (−0.24)

Loadings are indicated in parentheses. See Extended Data Tables 1-1–1-5 for expanded lists of top, middle, and bottom 10 stimulus model loadings.

10.1523/JNEUROSCI.1318-24.2024.f6-1 Figure 6-1 Top, middle, and bottom ten object concepts (within training set) scored along PLSR Component 1. Middle scores are defined as the bottom five above and top five below zero. Example images are illustrated for each concept, and scores are indicated in parentheses. Images in this figure have been replaced with images from the THINGSplus dataset distributed under a CC0 licence, which are representative of each object concept. Download Figure 6-1, TIF file.

10.1523/JNEUROSCI.1318-24.2024.f6-2 Figure 6-2 Top, middle, and bottom ten object concepts (within training set) scored along PLSR Component 2. Middle scores are defined as the bottom five above and top five below zero. Example images are illustrated for each concept, and scores are indicated in parentheses. Images in this figure have been replaced with images from the THINGSplus dataset distributed under a CC0 licence, which are representative of each object concept. Download Figure 6-2, TIF file.

10.1523/JNEUROSCI.1318-24.2024.f6-3 Figure 6-3 Top, middle, and bottom ten object concepts (within training set) scored along PLSR Component 3. Middle scores are defined as the bottom five above and top five below zero. Example images are illustrated for each concept, and scores are indicated in parentheses. Images in this figure have been replaced with images from the THINGSplus dataset distributed under a CC0 licence, which are representative of each object concept. Download Figure 6-3, TIF file.

10.1523/JNEUROSCI.1318-24.2024.f6-4 Figure 6-4 Top, middle, and bottom ten object concepts (within training set) scored along PLSR Component 4. Middle scores are defined as the bottom five above and top five below zero. Example images are illustrated for each concept, and scores are indicated in parentheses. Images in this figure have been replaced with images from the THINGSplus dataset distributed under a CC0 licence, which are representative of each object concept. Download Figure 6-4, TIF file.

10.1523/JNEUROSCI.1318-24.2024.f6-5 Figure 6-5 Top, middle, and bottom ten object concepts (within training set) scored along PLSR Component 5. Middle scores are defined as the bottom five above and top five below zero. Example images are illustrated for each concept, and scores are indicated in parentheses. Images in this figure have been replaced with images from the THINGSplus dataset distributed under a CC0 licence, which are representative of each object concept. Download Figure 6-5, TIF file.

10.1523/JNEUROSCI.1318-24.2024.t1-1 Table 1-1 Top, middle, and bottom ten stimulus model dimensions loading on PLSR Component 1. Middle loadings are defined as the bottom five above and top five below zero. Loadings are indicated in parentheses. Download Table 1-1, DOCX file.

10.1523/JNEUROSCI.1318-24.2024.t1-2 Table 1-2 Top, middle, and bottom ten stimulus model dimensions loading on PLSR Component 2. Middle loadings are defined as the bottom five above and top five below zero. Loadings are indicated in parentheses. Download Table 1-2, DOCX file.

10.1523/JNEUROSCI.1318-24.2024.t1-3 Table 1-3 Top, middle, and bottom ten stimulus model dimensions loading on PLSR Component 3. Middle loadings are defined as the bottom five above and top five below zero. Loadings are indicated in parentheses. Download Table 1-3, DOCX file.

10.1523/JNEUROSCI.1318-24.2024.t1-4 Table 1-4 Top, middle, and bottom ten stimulus model dimensions loading on PLSR Component 4. Middle loadings are defined as the bottom five above and top five below zero. Loadings are indicated in parentheses. Download Table 1-4, DOCX file.

10.1523/JNEUROSCI.1318-24.2024.t1-6 Table 1-6 Top, middle, and bottom ten stimulus model dimensions loading on PLSR Component 5. Middle loadings are defined as the bottom five above and top five below zero. Loadings are indicated in parentheses. Download Table 1-6, DOCX file.

**Figure 7.**
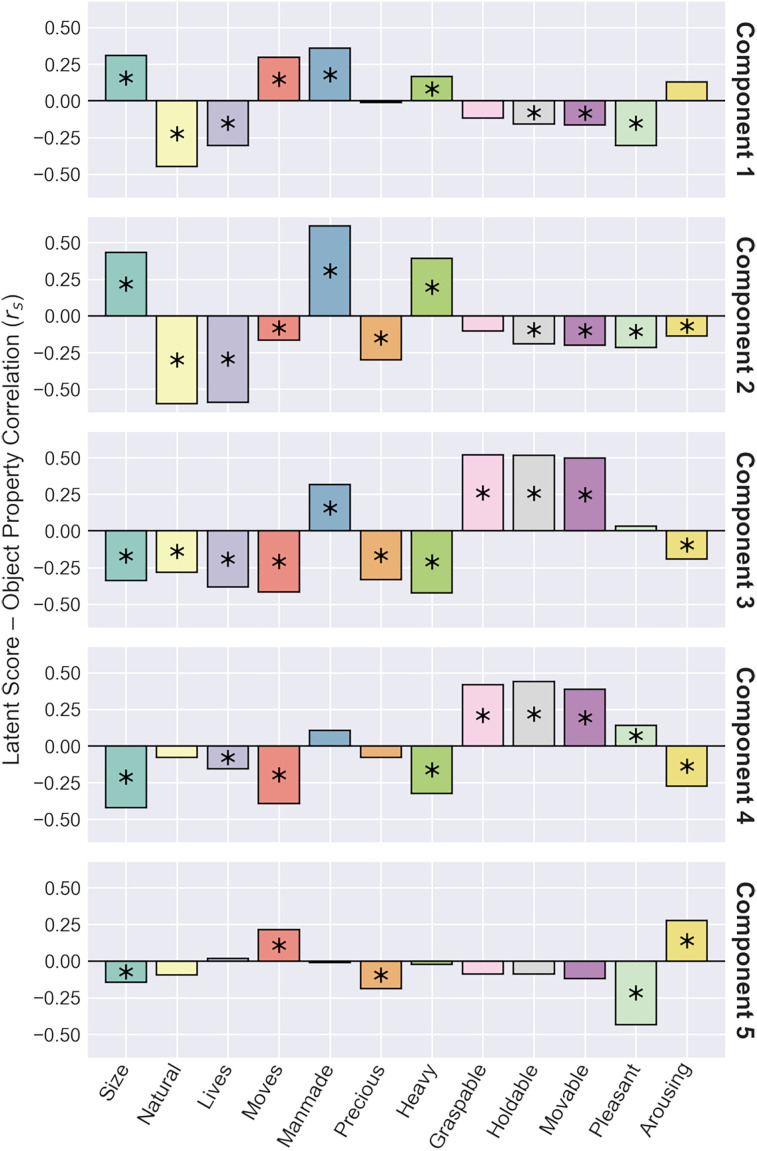
Spearman correlations between latent scores on each component and 12 object property ratings from the THINGSplus metadata for object concepts in the training set. Bars marked with asterisks indicate significant correlations (*p *< 0.05, FWER corrected). Extended Data Figure 7-1 illustrates correlations between object properties.

To provide additional descriptive details, we obtained ratings for the object concepts in the training set along 12 object properties included in the THINGSplus metadata (Stoinski et al., 2023). Figure 7 illustrates correlations between ratings on the 12 object properties and latent scores along the five PLSR components. Extended Data Figure 7-1 illustrates correlations between the object properties themselves.

10.1523/JNEUROSCI.1318-24.2024.f7-1 Figure 7-1 (a) Spearman correlations between 12 object property ratings from the THINGSplus metadata for object concepts in the training set. Additional visualisations are provided by (b) hierarchical clustering and (c) multidimensional scaling analyses of correlation distances. Download Figure 7-1, TIF file.

The first component was most positively associated with various body- and face-related objects concepts (Table 1, Fig. 6). However, comparison with the object property ratings (Fig. 7) suggested the component as a whole was more generally positively associated with manmade objects, as well as large, moving, and heavy objects. Meanwhile, the component was negatively associated with highly textured images—particularly those related to food and plants (Table 1, Fig. 6). It was also negatively correlated with natural, living, holdable, movable, and pleasant object properties (Fig. 7*c*). This accords with the anterior-posterior representation in the brain: highly textured images are likely to drive a strong response in early visual regions, while more tangible objects are associated with category-selective responses in ventral visual regions.

The second component was positively associated with larger artificial object concepts (Table 1, Fig. 6) and similarly was positively correlated with large, manmade, and heavy object properties (Fig. 7). Meanwhile, it was negatively associated with smaller natural objects—particularly those related to faces and bodies (Table 1, Fig. 6). Similarly, it was negatively correlated with natural, living, and moving object properties, as well as precious, holdable, movable, pleasant, and arousing objects (Fig. 7). This is also consistent with the medial-lateral neural representation, where positive loadings overlapped medial scene-selective regions, while negative loadings overlapped more lateral regions often associated with selectivity for animate objects.

The third component was positively associated with small and elongated objects, indicating it may embody visual features relating to size and shape. However, it was also negatively associated with animals, potentially also suggesting a representation of animacy (Table 1, Fig. 6). Comparisons with the object property ratings indicated positive correlations with manmade, graspable, holdable, and moveable objects and negative correlations with large, natural, living, moving, precious, heavy, and arousing objects (Fig. 7).

The fourth component was often negatively associated with objects related to fire or explosions and positively associated with small objects, but not exclusively so (Table 1, Fig. 6). Comparison with the object property ratings (Fig. 7) indicates the component was positively correlated with graspable, holdable, moveable, and pleasant objects and negatively correlated with large objects, indicating a possible positive association with manipulable objects. It was further negatively correlated with moving, heavy, and arousing objects, perhaps suggesting a negative association with more semantic features such as how exciting or stimulating an object is.

Finally, the fifth component was positively associated with thin and spindly or stick-shaped objects (Table 1, Fig. 6), suggesting a representation that may include shape features. It was also positively correlated with moving and arousing object properties (Fig. 7). The component was negatively associated with larger objects (Table 1, Fig. 6), suggesting representations including size and shape features. Comparison with the object property ratings (Fig. 7) similarly indicated a negative correlation with large objects, but also with precious and pleasant objects, suggesting possible additional representations of higher-level semantic features.

### Representational similarity between PLSR components and artificial neural networks

Next, we compared the feature representations within the PLSR components to representations found in DCNNs that have been pretrained to discriminate objects in the ImageNet set. To make this comparison, we conducted a series of RSAs (Kriegeskorte et al., 2008a), in which the PLSR latent scores were compared with layer activations in AlexNet and VGG16 DCNNs. Images for each of the 480 object concepts in the training set were passed through the DCNNs. Activations were extracted for each of the convolutional and fully connected layers and averaged over images within each concept. DCNN RDMs were constructed for each layer by computing pairwise correlation distances between the object concepts.

**Figure 8.**
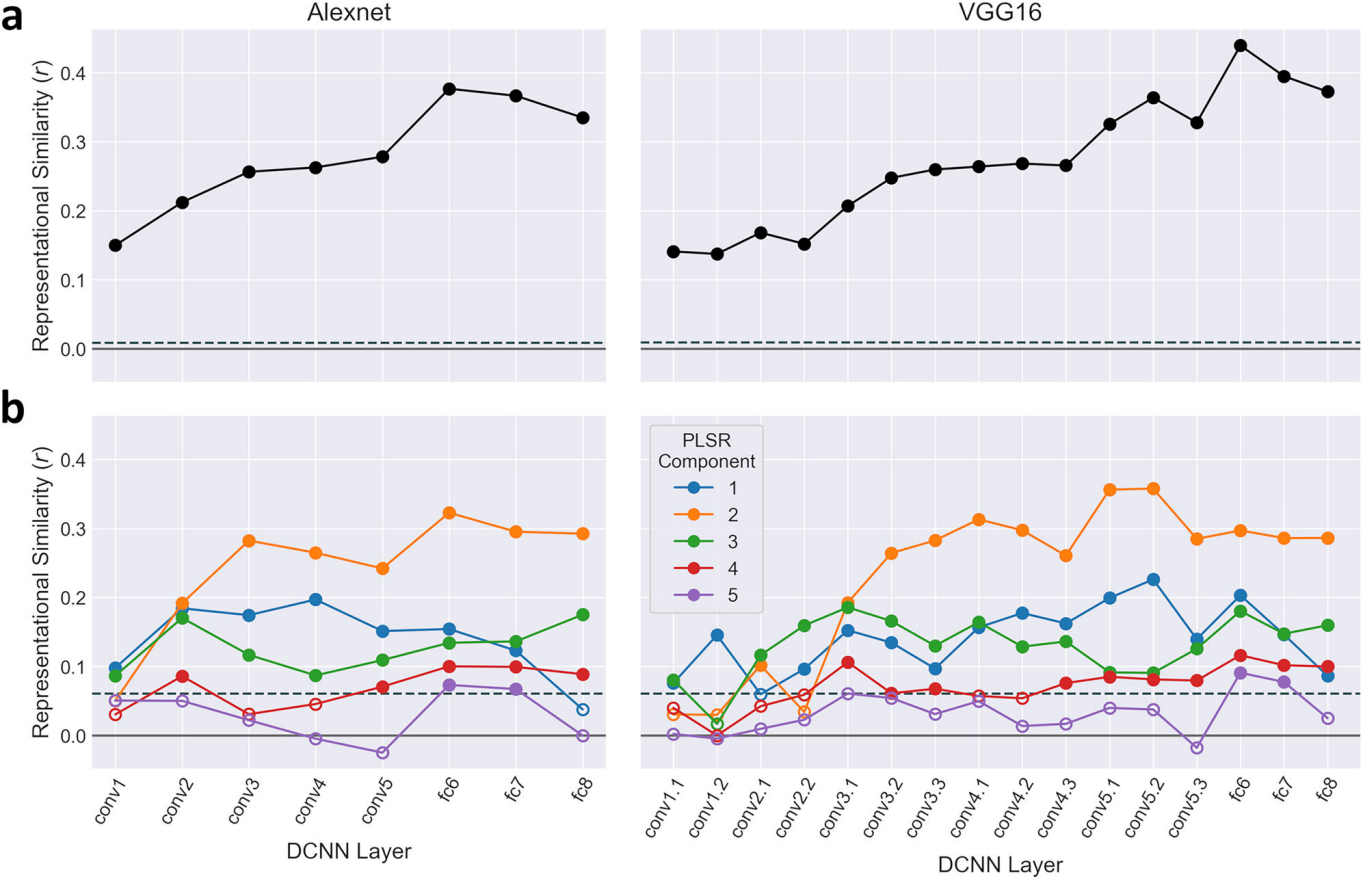
Representational similarity analyses between PLSR and DCNN representations of object concepts in the training set. Representational dissimilarity was measured from DCNN layer activations to images from each object concept using two DCNNs trained for object identification: AlexNet and VGG16. These were then correlated with corresponding measures of representational dissimilarity based on the PLSR latent scores. Representational dissimilarity of PLSR latent scores was estimated for (***a***) the full five-dimensional latent space and (***b***) each component separately. Filled symbols indicate significant correlations and dashed lines indicate significance thresholds (*p *< 0.05; FWER corrected), as determined by permutation testing.

Next, we computed an RDM for the full PLSR latent space by measuring pairwise correlation distances between object concepts using their five-dimensional latent scores. RSAs were then performed correlating this PLSR RDM against the RDMs for each DCNN layer (Fig. 8*a*). The results were consistent between the AlexNet and VGG16 networks: significant correlations were observed between the PLSR and DCNN representations for all layers but with correlations generally increasing across the convolutional layers and peaking within the earliest fully connected layer (fc6). Correlations then decreased, though remained high, for the later fully connected layers (fc7 and fc8). This suggests the PLSR representations are best captured by mid- to high-level features of the objects.

We also performed RSAs for each of the PLSR components independently. PLSR RDMs were constructed by calculating the pairwise absolute difference in scores between object concepts for each component separately, which were then correlated with the DCNN RDMs for each layer (Fig. 8*b*). In general, greater representational similarity was observed for later convolutional and early fully connected layers than for early convolutional or later fully connected layers. Component 2 showed the highest representational similarity across later DCNN layers, followed by components 1 and 3, while the lowest similarity was observed for components 4 and 5.

### Comparison of PLSR and linear encoding models

**Figure 9.**
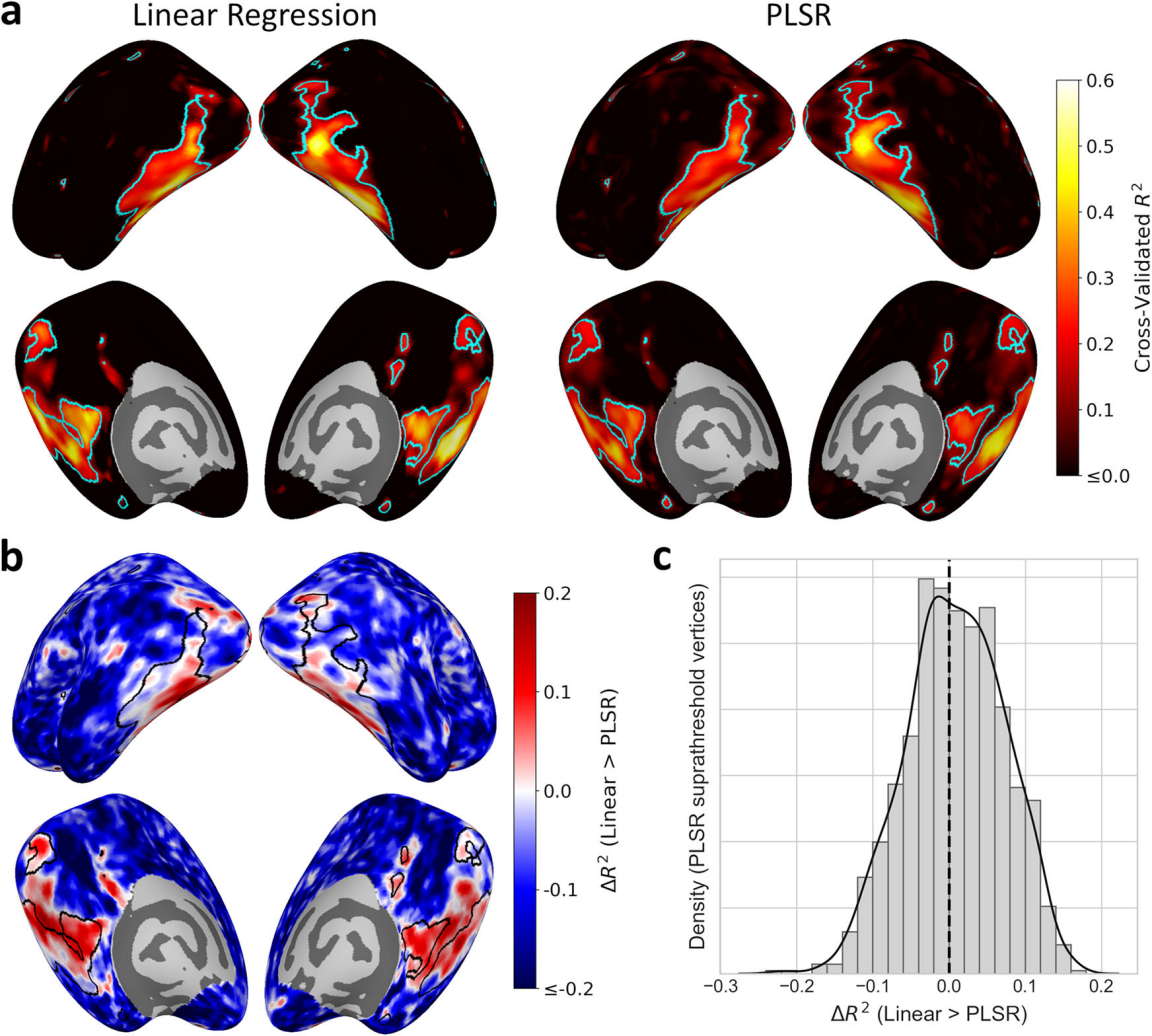
Comparison of linear regression encoding model, using all 66 stimulus features, to PLSR encoding model using five components. ***a***, Cross-validated *R*^2^ maps showing the variance explained in univariate responses across object concepts in the test set for the linear regression and PLSR models. ***b***, Difference in *R*^2^ maps between the encoding models (linear regression > PLSR). Cyan/black lines indicate significance threshold for PLSR *R*^2^ map (*p *< 0.05, FWER corrected; compare Fig. 1*b*). ***c***, Distribution of *R*^2^ differences values over vertices above the PLSR *R*^2^ significance threshold. Despite using fewer dimensions, the PLSR only incurred a small loss of explained variance in the visual cortex compared with the linear regression model.

While the PLSR is able to predict neural responses using a small number of components, it is possible that the dimensionality reduction may also miss key sources of variance in the response. To this end, we compared the PLSR model to a standard linear regression that maps the 66 stimulus features directly to the neural responses without applying any dimensionality reduction. The model was again fit to the 480 object concepts in the training set and tested on the 240 held-out concepts. Figure 9*a* illustrates the distribution of cross-validated *R*^2^ values across the brain for both models. Both the linear and PLS models highlight similar brain regions, with the highest performance observed in early and ventral visual cortices (up to ∼59% variance explained by the linear model). Figure 9*b* illustrates the difference between the *R*^2^ maps. The linear model explains more variance than the PLSR throughout much of high-level visual cortex. Nevertheless, despite the PLSR reducing the number of predictors from 66 down to just five, the loss of explained variance is only small. Furthermore, the PLSR outperforms the linear model outside of visual regions, indicating the linear model is more prone to overfitting in regions with noisier responses. Figure 9*c* illustrates the distribution of *R*^2^ difference values for vertices above the significance threshold for the PLSR *R*^2^ map (compare Fig. 1*b*). Although the linear encoding model outperforms PLSR in some vertices, the differences are negligible or even indicate better performance for the PLSR model among other vertices. Thus, although the PLSR does incur a loss of explained variance in high-level visual regions, the magnitude of this loss is relatively small considering the substantial reduction in dimensionality. Furthermore, the full linear model does not highlight additional brain regions beyond those already well predicted by the PLSR.

## Discussion

We employed a data-driven approach to reveal key organizing principles governing the representation of natural objects in the human brain. Using data from the THINGS initiative (Hebart et al., 2019, 2023), we constructed a neural encoding model based on PLSR which identified a small number of components explaining the neural response to objects. These components exhibited continuous graded representations across the cortical surface and bilateral symmetry. They also encoded complex combinations of stimulus features yet did not straightforwardly correspond to previous explanations of the organization of the visual cortex. Instead, our results indicate the neural representation of objects is driven by a statistically efficient encoding of the visual environment.

A significant challenge in identifying organizing principles within high-level visual cortex is the limited number of images typically presented in neuroimaging experiments, which represent only a fraction of the stimuli encountered during natural viewing. Moreover, images are often assigned to conditions defined by the experimenter, potentially constraining interpretations to preconceived notions on the organization of the visual cortex. To address this, we employed stimulus features derived from a data-driven analysis of perceptual similarity judgments across a large and diverse set of natural objects (Hebart et al., 2019, 2020). We then applied PLSR to predict neural responses from these stimulus features while simultaneously reducing dimensionality. Using only five components, the PLSR accurately predicted responses throughout low- and high-level visual cortex, with the highest accuracies (explaining up to almost 50% of the variance) observed in the ventral visual stream. The neural loadings of each component revealed graded, continuous representations over the cortical surface which were similar in each hemisphere. A follow-up GLM analysis confirmed these topographic patterns were reproducible for objects withheld from the initial PLSR fitting. Thus, the PLSR identified continuous topographic representations that accurately predicted neural responses across the visual brain and were consistent across hemispheres and data partitions. Importantly, the graded nature of the representations and the bilateral symmetry were not imposed by the PLSR itself, as the model could have produced sparse or distributed solutions.

Each component exhibited distinct representations across the cortical surface. The first component revealed a posterior-anterior gradient distinguishing between low- and high-level visual regions, while the second component indicated a medial-lateral gradient spanning the ventral stream. Subsequent components revealed repeating patterns along both medial-lateral and anterior-posterior axes, analogous to the reversing topographic maps observed in primary sensory regions of the brain (Formisano et al., 2003; Sanchez-Panchuelo et al., 2010; Wandell and Winawer, 2011). Many components identified stimulus features according with previous descriptions of the ventral stream, such as object size (Konkle and Oliva, 2012), animacy (Chao et al., 1999; Kriegeskorte et al., 2008b; Konkle and Caramazza, 2013), rectilinearity (Nasr et al., 2014), and shape (Bao et al., 2020; Coggan and Tong, 2023). A key advance from our approach is that these features emerge from a data-driven analysis of diverse natural objects. However, the components often combined stimulus features in ways that did not directly align with previous models of object recognition. For instance, many components encoded multiple features rather than isolating distinct maps for individual properties. The components suggest neural representations in the visual cortex are optimized to efficiently encode natural variation in object statistics. While these representations capture essential aspects of object perception, they need not necessarily lend themselves to straightforward interpretations.

PLSR optimizes the components to explain variance in the outcome variables, thereby maximizing prediction accuracy while reducing dimensionality (Krishnan et al., 2011). In contrast, traditional neural encoding approaches may employ anywhere between dozens to thousands of predictors, depending on the complexity of the stimulus model (Naselaris et al., 2011). Recently, Contier et al. (2024) applied a linear encoding model to the THINGS database, using the same 66 behaviorally derived dimensions utilized in the present study to explain neural responses. They revealed sparse and distributed representations of these stimulus dimensions. In contrast, our PLSR model retained only five latent components yet still predicted responses extensively across the visual cortex with a high level of accuracy. Despite the low dimensionality, PLSR only incurred a slight loss of prediction accuracy compared with a linear encoding model which did not incorporate any dimensionality reduction, with both models yielding high accuracies in similar brain regions. This suggests PLSR offers a more parsimonious framework for understanding the organization of the visual brain by identifying a low-dimensional representation of object properties which still accurately predicts neural responses.

Previous studies have employed dimensionality reduction alongside neural encoding by using linear encoding to map stimulus features directly to neural responses, then applying PCA to decompose the high-dimensional regression coefficients. Such approaches have yielded interpretable components corresponding to key semantic features (Huth et al., 2012, 2016). Nevertheless, PLSR differs from such approaches in two key ways. First, principal components representing correlations between regression coefficients do not directly correspond to the prediction accuracy of the encoding model nor is there any guarantee they will capture the maximal sources of variation in the neural response. In contrast, PLSR components are explicitly defined by their predictive power; hence model predictions directly reflect the components selected. Second, because linear regression coefficients only describe the relationship between the dimensions of the stimulus and neural spaces, information about the samples is lost. Conversely, by inspecting the PLSR loadings and latent scores, it is possible to relate the components to the samples as well as the stimulus and neural dimensions. Indeed, the latent scores of the object concepts in the PLSR model were highly informative of the properties represented by each component.

Several studies have utilized data-driven approaches to investigate the organization of high-level visual cortex. These studies have reproduced classic category-selective responses (Haxby et al., 2011; Vul et al., 2012), while also identifying novel responses such as to images of food (Khosla et al., 2022). We previously employed data-driven approaches for stimulus selection, demonstrating the critical role of visual features in the organization of the ventral visual stream (Watson et al., 2017; Coggan et al., 2019). However, a limitation of applying data-driven analyses to only one domain—whether brain responses, stimulus properties, or behavior—is that they do not provide an explicit link between domains. For instance, dimensions derived entirely from visual properties are not guaranteed to be perceptually relevant. A key advance of the current approach is to directly link behaviorally relevant stimulus features to neural representations in a fully data-driven framework. This allows a more comprehensive understanding of how perceptual and neural representations interact, offering insights going beyond a single domain.

The stimulus dimensions used in this study have the advantage of being data-driven, behaviorally relevant and encompass a wide range of low-, mid-, and high-level object features (Hebart et al., 2020, 2023). These dimensions were defined at the level of object concepts, rather than individual images. Although images within object concepts will show similar stimulus features, future research may benefit from incorporating image-level variation (Contier et al., 2024). Moreover, alternative neural representations may be captured by additional stimulus features not included in the current analysis, such as low-level visual properties (Kay et al., 2008; Rice et al., 2014) or higher-level semantic attributes (Huth et al., 2012). The flexibility of the PLSR approach is well suited to other stimulus features or even entirely different modalities, offering a versatile tool for exploring diverse aspects of neural representation.

DCNNs have increasingly been used as computational models for understanding object perception. DCNNs often produce neural representations aligned with the human visual system (Khaligh-Razavi and Kriegeskorte, 2014; Cichy et al., 2016; Simony et al., 2024). In the present study, we compared representations within the PLSR latent space to two widely used DCNNs (AlexNet and VGG16) trained for object identification. Our results demonstrated significant representational similarity with all DCNN layers, with the highest similarity observed in later convolutional and early fully connected layers. This suggests the PLSR components represent a range of low-, mid-, and high-level object features. Furthermore, latent scores along each component correlated with behavioral ratings of key object properties included in the THINGSplus metadata (Stoinski et al., 2023). Thus, the PLSR components embodied key object properties and features relevant for object identification.

In conclusion, we implemented a data-driven approach to predict neural responses to natural objects from their perceptual properties. A neural encoding model using PLSR predicted neural responses from stimulus dimensions derived from a data-driven analysis of behavioral similarity judgments, thus avoiding experimenter-defined preconceptions of underlying stimulus features. The model accurately predicted neural responses across numerous regions of the visual cortex using only a small number of components. These components revealed smooth, graded topographic neural representations that were similar in each hemisphere and captured various object properties including animacy, real-world size, and category. Importantly, these representations did not align in any simple way with existing theoretical perspectives on object perception. Instead, our findings suggest the visual brain encodes information in a statistically efficient way, reflecting natural variability among objects.

## Data Availability

The raw behavioral and MRI data are already made publicly available by the THINGS initiative (https://things-initiative.org/). Our analysis code and data outputs are available on the Open Science Framework (https://osf.io/scy2f/).
