# Supplementary figures and images for: A Data-Driven Analysis of the Perceptual and Neural Responses to Natural Objects Reveals Organizing Principles of Human Visual Cognition
Source: J Neurosci. 2024 Nov 18;45(2):e1318242024. doi: 10.1523/JNEUROSCI.1318-24.2024 (PMC11714349; doi:10.1523/JNEUROSCI.1318-24.2024)

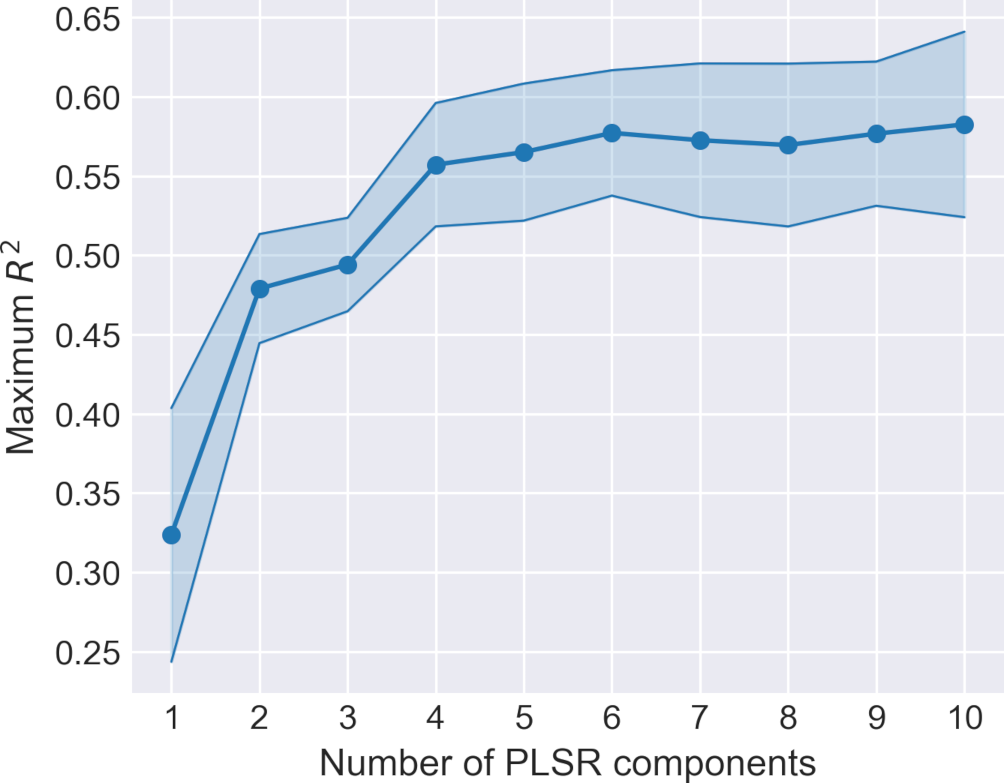

Supplement: Figure 1-1 — Grid search of prediction accuracy over varying numbers of PLSR components to retain. A 5-fold cross-validation was nested within the 480 object concepts of the main training set. For each fold of the cross-validation, a PLSR model was fit to the inner training set, retaining between 1 and 10 components, then whole-brain R2 maps were calculated for the inner test set. A summary statistic was calculated by taking the maximum R2 value over all vertices, indicating the peak prediction accuracy over the whole brain. The graph illustrates the mean and standard deviation (over the 5 cross-validation folds) of these maximal R2 values for each number of components. Prediction accuracy plateaus between 4 and 6 components. Download Figure 1-1, TIF file. [file jneuro-45-e1318242024-s006.tif]

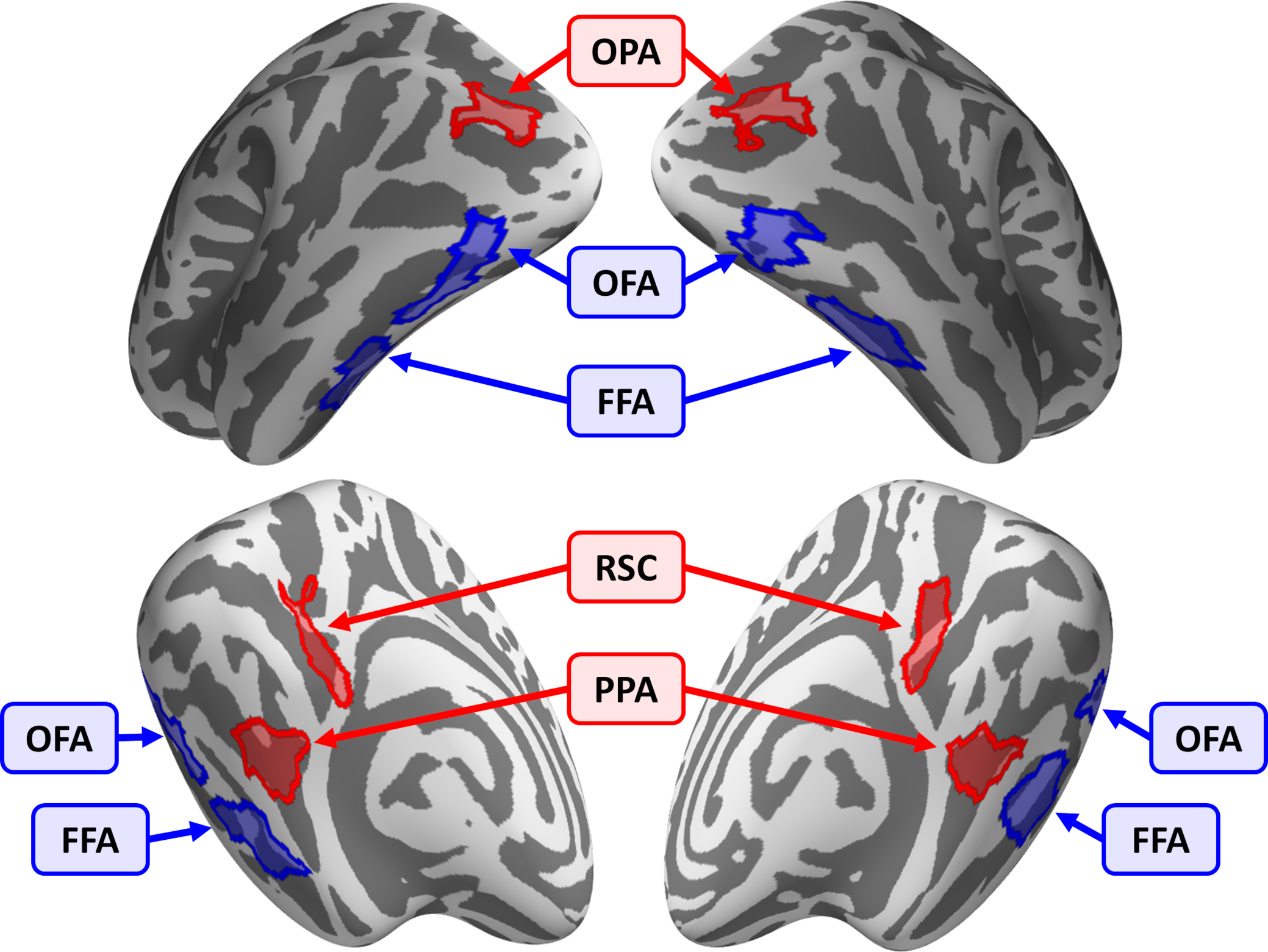

Supplement: Figure 1-2 — Locations of core scene- and face-selective regions of interest. Regions are defined from the group average contrast of “scenes > faces” using the category localiser task data. Scene regions: Parahippocampal Place Area (PPA), Retrosplenial Complex (RSC), Occipital Place Area (OPA). Face regions: Fusiform Face Area (FFA), Occipital Face Area (OFA). Download Figure 1-2, TIF file. [file jneuro-45-e1318242024-s007.tif]

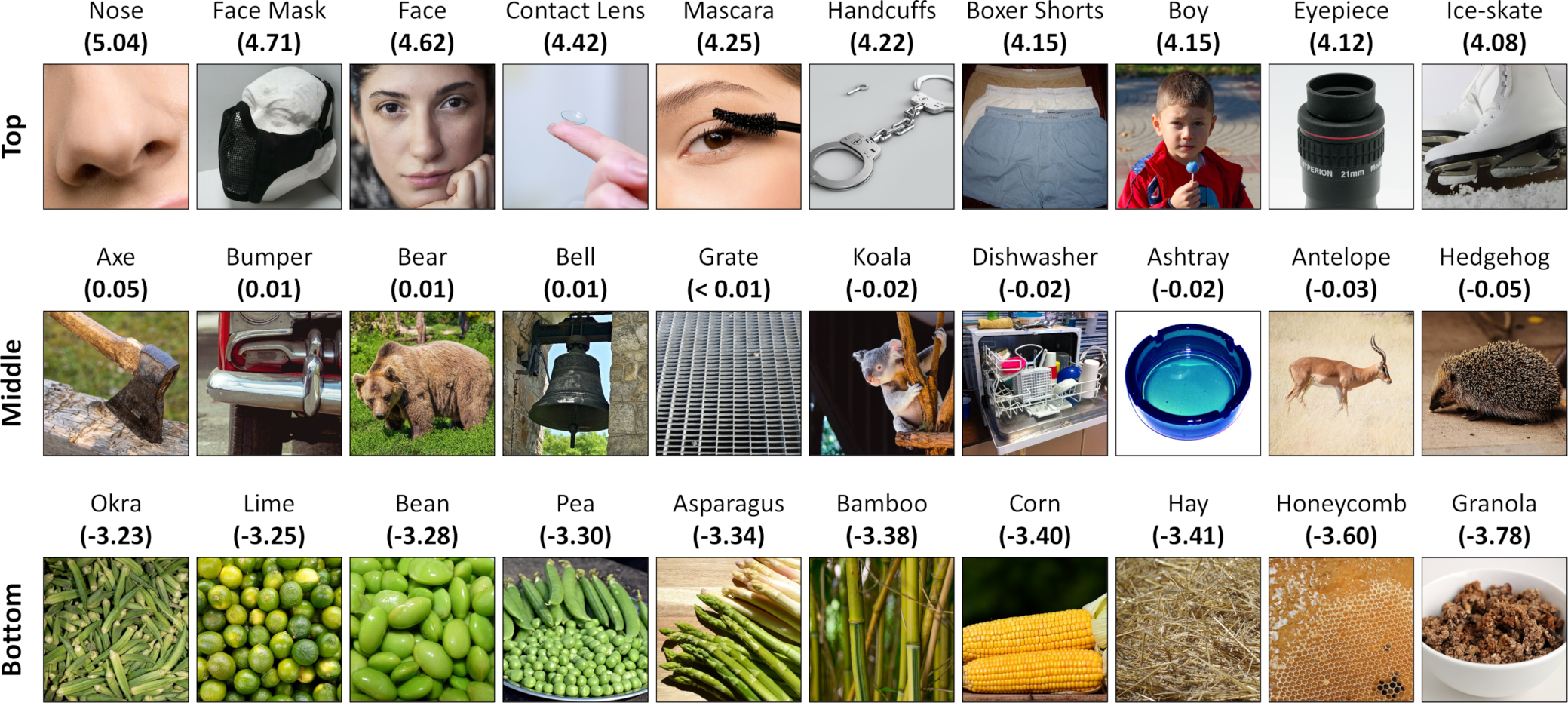

Supplement: Figure 6-1 — Top, middle, and bottom ten object concepts (within training set) scored along PLSR Component 1. Middle scores are defined as the bottom five above and top five below zero. Example images are illustrated for each concept, and scores are indicated in parentheses. Images in this figure have been replaced with images from the THINGSplus dataset distributed under a CC0 licence, which are representative of each object concept. Download Figure 6-1, TIF file. [file jneuro-45-e1318242024-s008.tif]

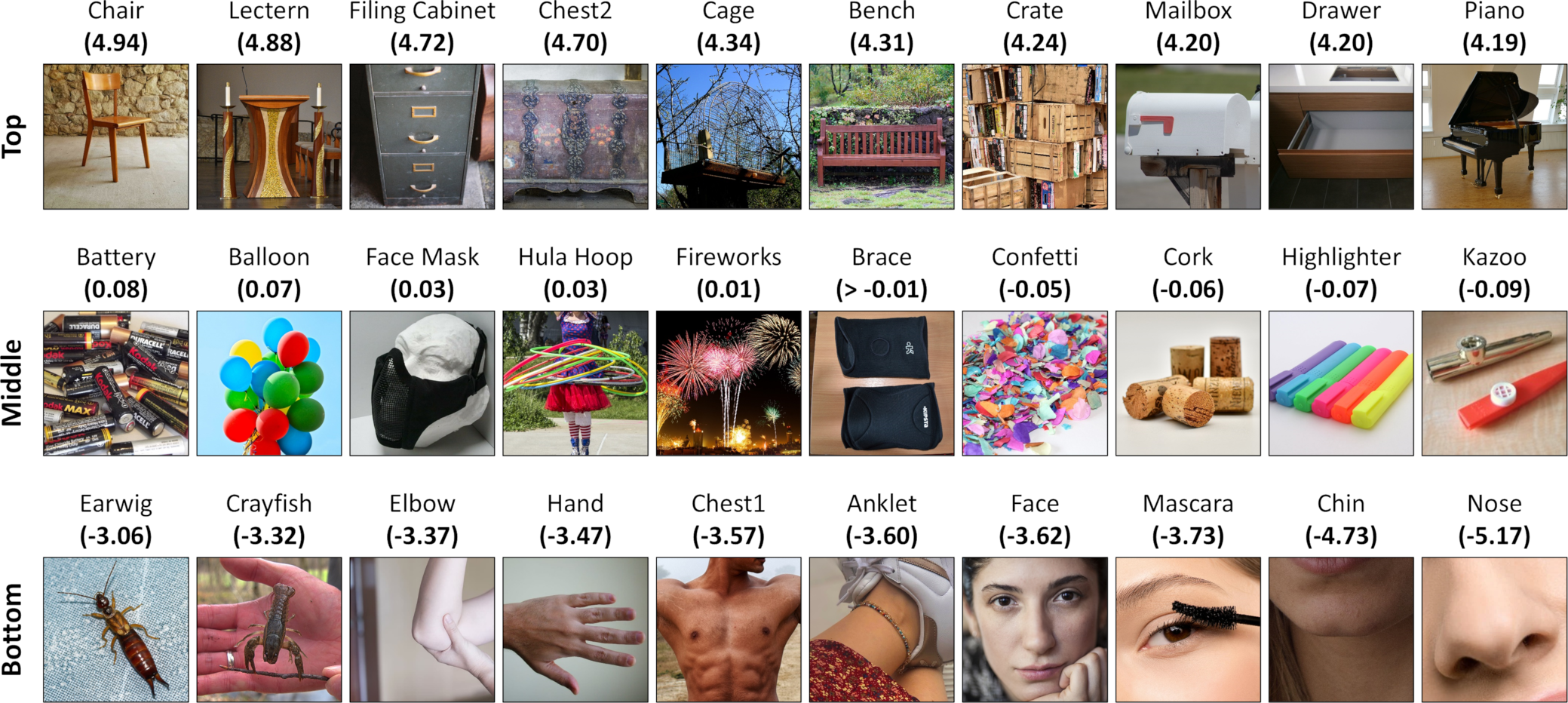

Supplement: Figure 6-2 — Top, middle, and bottom ten object concepts (within training set) scored along PLSR Component 2. Middle scores are defined as the bottom five above and top five below zero. Example images are illustrated for each concept, and scores are indicated in parentheses. Images in this figure have been replaced with images from the THINGSplus dataset distributed under a CC0 licence, which are representative of each object concept. Download Figure 6-2, TIF file. [file jneuro-45-e1318242024-s009.tif]

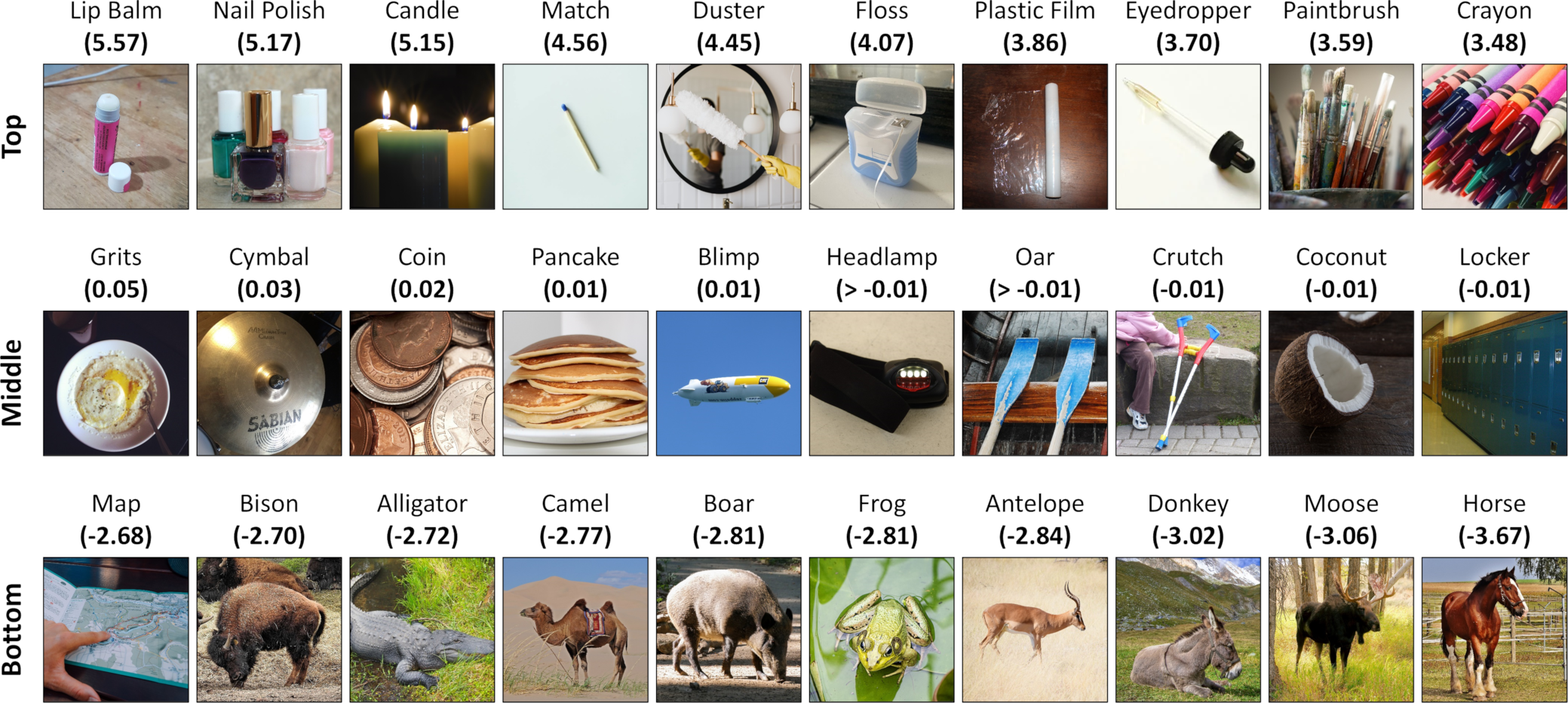

Supplement: Figure 6-3 — Top, middle, and bottom ten object concepts (within training set) scored along PLSR Component 3. Middle scores are defined as the bottom five above and top five below zero. Example images are illustrated for each concept, and scores are indicated in parentheses. Images in this figure have been replaced with images from the THINGSplus dataset distributed under a CC0 licence, which are representative of each object concept. Download Figure 6-3, TIF file. [file jneuro-45-e1318242024-s010.tif]

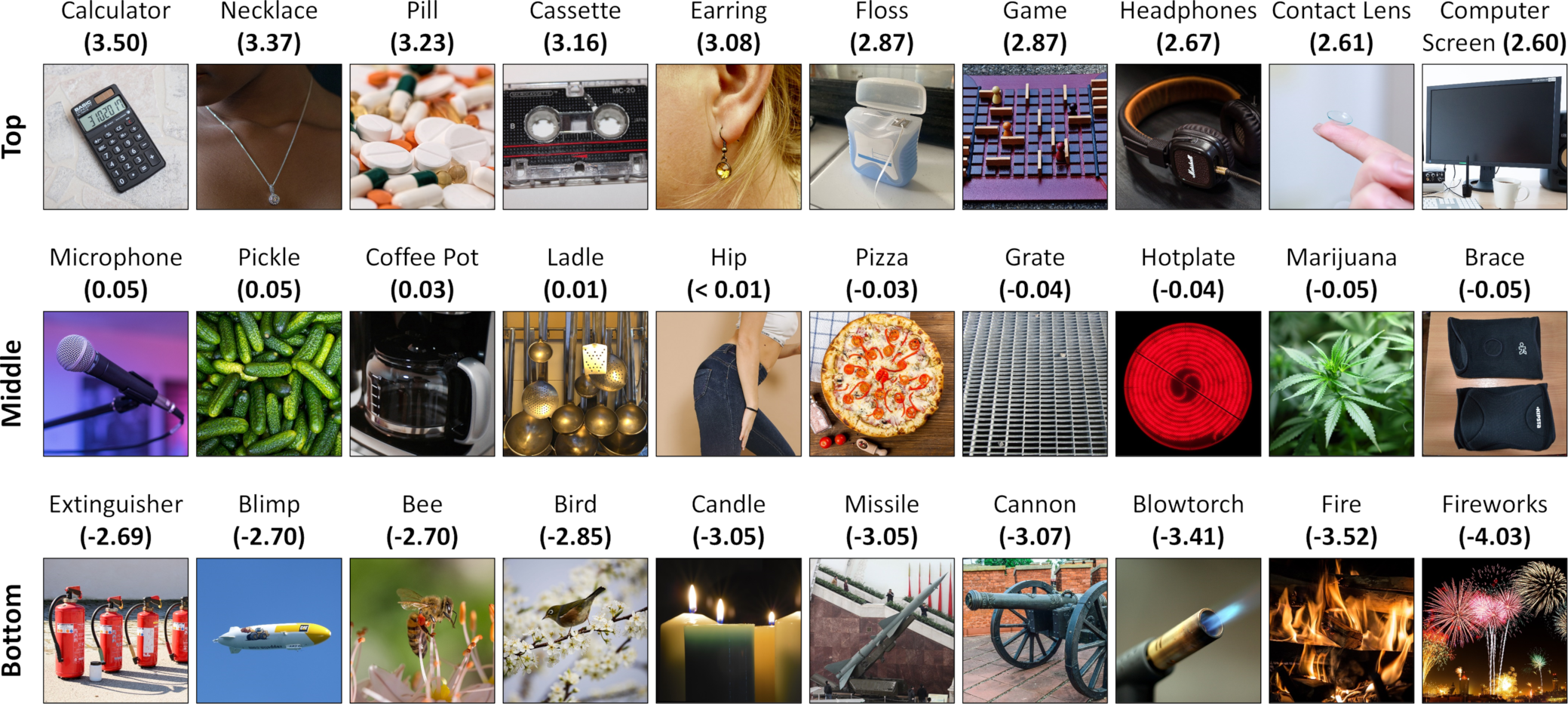

Supplement: Figure 6-4 — Top, middle, and bottom ten object concepts (within training set) scored along PLSR Component 4. Middle scores are defined as the bottom five above and top five below zero. Example images are illustrated for each concept, and scores are indicated in parentheses. Images in this figure have been replaced with images from the THINGSplus dataset distributed under a CC0 licence, which are representative of each object concept. Download Figure 6-4, TIF file. [file jneuro-45-e1318242024-s011.tif]

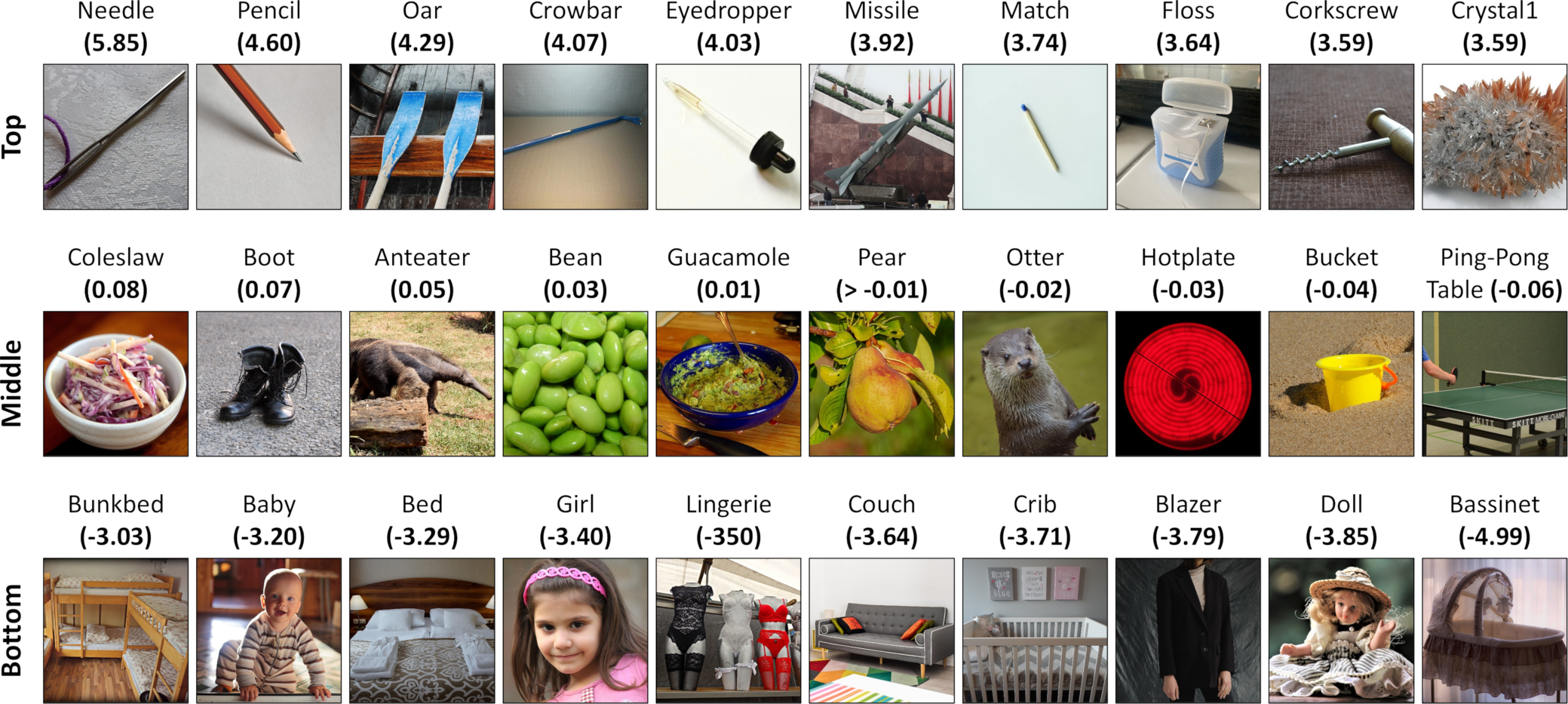

Supplement: Figure 6-5 — Top, middle, and bottom ten object concepts (within training set) scored along PLSR Component 5. Middle scores are defined as the bottom five above and top five below zero. Example images are illustrated for each concept, and scores are indicated in parentheses. Images in this figure have been replaced with images from the THINGSplus dataset distributed under a CC0 licence, which are representative of each object concept. Download Figure 6-5, TIF file. [file jneuro-45-e1318242024-s012.tif]

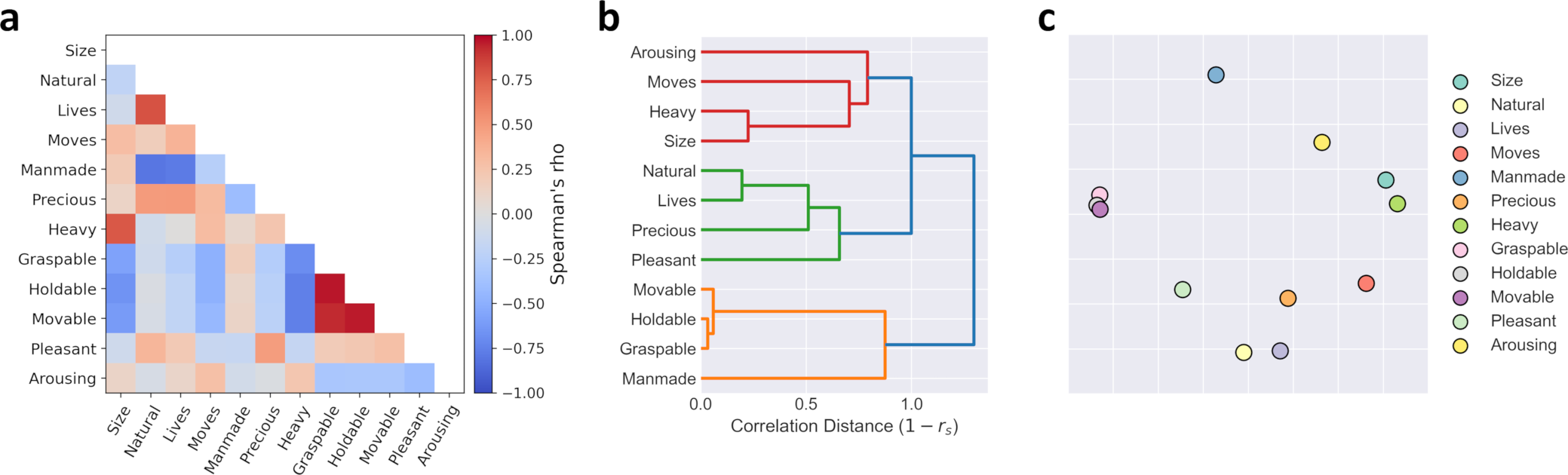

Supplement: Figure 7-1 — (a) Spearman correlations between 12 object property ratings from the THINGSplus metadata for object concepts in the training set. Additional visualisations are provided by (b) hierarchical clustering and (c) multidimensional scaling analyses of correlation distances. Download Figure 7-1, TIF file. [file jneuro-45-e1318242024-s013.tif]
