# Supplementary material for: A Data-Driven Analysis of the Perceptual and Neural Responses to Natural Objects Reveals Organizing Principles of Human Visual Cognition
Source: J Neurosci. 2024 Nov 18;45(2):e1318242024. doi: 10.1523/JNEUROSCI.1318-24.2024 (PMC11714349; doi:10.1523/JNEUROSCI.1318-24.2024)
Supplement: Table 1-2 — Top, middle, and bottom ten stimulus model dimensions loading on PLSR Component 2. Middle loadings are defined as the bottom five above and top five below zero. Loadings are indicated in parentheses. Download Table 1-2, DOCX file. [file jneuro-45-e1318242024-s002.docx]

| **Table 1-2.** Top, middle, and bottom ten stimulus model dimensions loading on PLSR Component 2. Middle loadings are defined as the bottom five above and top five below zero. Loadings are indicated in parentheses. | | |
| --- | --- | --- |
| **Top** | **Middle** | **Bottom** |
| Metallic/artificial **(0.33)** | Pointed/spiky **(0.01)** | Green **(-0.08)** |
| House-related/furnishing-related **(0.33)** | Colourful/playful **(0.01)** | Feminine (stereotypical) **(-0.09)** |
| Box-related/container **(0.25)** | Thin/flat/wrapping **(0.01)** | Spherical/voluminous **(-0.11)** |
| Seating-/standing-/lying-related **(0.24)** | Bathroom-/wetness-related  **(< 0.01)** | Sweet / dessert-related **(-0.11)** |
| Wood-related/brown **(0.22)** | String-related/curved **(< 0.01)** | Bug-related/non-mammalian/disgusting **(-0.15)** |
| Electronics/technology **(0.22)** | Yellow **(-0.01)** | Plant-related **(-0.17)** |
| Upright/elongated/voluminous **(0.21)** | Tubular **(-0.01)** | Body-/people-related  **(-0.17)** |
| Transportation-/movement-related **(0.2)** | Transparent/shiny/crystalline  **(-0.01)** | Food-related **(-0.19)** |
| Beams-/mesh-related **(0.19)** | Decay-related/grainy **(-0.01)** | Body part-related **(-0.21)** |
| Paper-related/flat **(0.16)** | Coldness-related/winter-related  **(-0.01)** | Animal-related **(-0.25)** |
